# Influence of Target Location, Size, and Patient Age on Normal Tissue Sparing- Proton and Photon Therapy in Paediatric Brain Tumour Patient-Specific Approach

**DOI:** 10.3390/cancers12092578

**Published:** 2020-09-10

**Authors:** Mikaela Dell’Oro, Michala Short, Puthenparampil Wilson, Chia-Ho Hua, Melissa Gargone, Thomas E. Merchant, Eva Bezak

**Affiliations:** 1 Cancer Research Institute, University of South Australia, Adelaide SA 5001, Australia; Michala.Short@unisa.edu.au (M.S.); Eva.Bezak@unisa.edu.au (E.B.); 2 Department of Radiation Oncology, Royal Adelaide Hospital, Adelaide SA 5000, Australia; Puthenparampil.Wilson@unisa.edu.au; 3 UniSA STEM, University of South Australia, Adelaide SA 5001, Australia; 4 Department of Radiation Oncology, St. Jude Children’s Research Hospital, Memphis, TN 38105, USA; Chia-Ho.Hua@stjude.org (C.-H.H.); Melissa.Gargone@stjude.org (M.G.); thomas.merchant@stjude.org (T.E.M.); 5 Department of Physics, University of Adelaide, Adelaide SA 5005, Australia

**Keywords:** paediatric, proton therapy, brain tumour, IMPT, IMRT, target size and location, paediatric age, normal tissue sparing, patient-specific

## Abstract

**Simple Summary:**

This manuscript investigates the latest proton and photon radiation delivery techniques and the delivered dose distribution dependence on age and brain tumour location for simulated paediatric patients. Brain tumors are the leading cause of cancer-related burden in childhood cancer survivors. Standard treatment regimens include radiotherapy, and whilst photon therapy is commonly prescribed, proton particles (where available) have been proven to reduce the risk of long-term illness and morbidities. Differences between the two modalities are not fully quantified in paediatric patients for various intracranial tumour sites or age. Ependymoma proton plans demonstrated greater dose reduction for the 9 vs. 13-year-old patients (pituitary gland *p* < 0.001). Whilst medulloblastoma proton plans achieved greater maximum dose sparing to optic structures (4.8–12.6 Gy optic chiasm), brainstem sparing was limited (~0.5 Gy). Understanding these differences may help clinicians estimate the benefit and improve referral across available centres.

**Abstract:**

Background: Proton radiotherapy produces superior dose distributions compared to photon radiotherapy, reducing side effects. Differences between the two modalities are not fully quantified in paediatric patients for various intracranial tumour sites or age. Understanding these differences may help clinicians estimate the benefit and improve referral across available centres. Our aim was to compare intensity-modulated proton therapy (IMPT) and intensity-modulated photon radiotherapy (IMRT) radiation doses for select paediatric intracranial tumours. Methods: IMPT and IMRT dose distributions for gender-matched paediatric cranial CT-datasets (ages 5, 9 and 13 years) were retrospectively calculated to simulate irradiation of supratentorial (ependymoma) and infratentorial (medulloblastoma) target volumes diameters (1–3 cm) and position (central and 1–2 cm shifts). Results: Clinical dosimetric objectives were achieved for all 216 treatment plans. Whilst infratentorial IMPT plans achieved greater maximum dose sparing to optic structures (4.8–12.6 Gy optic chiasm), brainstem sparing was limited (~0.5 Gy). Mean dose difference for optic chiasm was associated with medulloblastoma target position (*p* < 0.0197). Supratentorial IMPT plans demonstrated greater dose reduction for the youngest patients (pituitary gland *p* < 0.001). Conclusions: Normal tissue sparing was achieved regardless of patient age for infratentorial tumours. However, for supratentorial tumours, there was a dosimetric advantage of IMPT across 9 vs. 13-year-old patients.

## 1. Introduction

The Australian Childhood Cancer Registry estimates that tumours of the central nervous system make up 25% of all diagnosed childhood cancers [1]. Due to their young age, paediatric patients’ therapeutic management is determined with the intention to preserve neurocognitive, growth, and endocrine function at this delicate stage of development [1,2]. Current radiotherapy management for paediatric brain tumours offers improved survival but carries risk of significant morbidity [3].

Proton therapy (PT) can reduce radiation-induced side effects as compared to photon therapy (XRT) [4]. PT exploits the charged nature of the proton, which deposits smaller amounts of energy along its path in normal tissue before it reaches its end of range, the Bragg peak, where majority of the energy deposition occurs. This unique dose deposition minimises radiation dose in the path proximal of the tumour and almost eliminates irradiation beyond, thus reducing normal tissue complication probability (NTCP) without decreasing optimal tumour control probability [5,6]. As PT significantly reduces the volume of healthy brain and critical structures irradiated with intermediate to low radiation doses compared to XRT, it has historically been employed for paediatric brain tumours. The decreased doses to organs at risk (OAR) result in fewer cognitive adverse effects (reported when doses exceed ≥18 Gy to the cranium), reducing developmental impairment [7,8]. Clinical outcomes including reduction in detrimental side effects of PT in paediatric patients have been highlighted in several publications [2,9,10,11,12]. However, the degree of dose reduction PT offers compared to XRT has not been well quantified by tumour location, tumour volume and patient age.

Majority of PT and XRT clinical data collected for paediatric patients is based on long-term studies conducted between 1963–2012; i.e., from a period involving older PT technologies. Similarly, recent meta-analysis reports on 3D conformal clinical studies and PT technology including passive scattering of the proton beam [9]. 3D conformal techniques, however, have now been superseded by the introduction of intensity-modulated photon radiotherapy (IMRT) and volumetric-modulated arc therapy (VMAT) for photons and by intensity-modulated proton therapy (IMPT) for protons. Pencil beam scanning technology has been progressively embraced in many centres compared to passive scattering over the last decade. Benefits of IMPT also include reduction of the lifetime attributable risk of developing second primary cancer resulting from high neutron production in the aperture and scatterers, used to produce clinical proton beams [13,14]. As scanning beam PT technology is still relatively new, there is minimal long-term clinical evidence regarding risks and benefits of this technology [12]. Literature shows that a latency period of up to 30 years would be required to observe a physical cancer incidence corresponding to a specific treatment type [15].

As long-term follow-ups and prospective studies are difficult to conduct in PT due to practical reasons, more consideration is given to retrospective and prospective planning comparative studies and modelling. In lieu of long-term clinical data, model-based approaches provide justification for adopting a new technology that has not yet produced adequate patient outcome data [16]. Although initial clinical results are promising, it is not well understood if PT should be the only RT modality to treat paediatric brain tumour patients.

This dosimetric study aims to provide information for selecting the patients who would benefit most from PT, as access to proton facilities may need to be prioritised. Previous retrospective comparative planning studies have not investigated the full dosimetric impact of tumour volume as a function of age whilst controlling for anatomical location and volume of the tumour. While a number of large-scale dosimetry studies have assessed several malignancies in order to estimate the benefits of PT, they range dramatically in terms of aim, variation of participants, and consistency of reporting [7,17,18,19]. Additionally, studies published to date did not consider dose-response variation with age, gender, or the target and the OAR radiosensitivity (α/β) [20].

Medulloblastoma (MB) and ependymoma are two of the most common paediatric brain tumours in children [21,22]. Radiotherapy plays a critical and unique role in the curative management of both tumour types. When clinically indicated, MB requires post-surgical neuraxis irradiation, which includes irradiation of the entire brain and spinal subarachnoid spaces commonly termed as craniospinal irradiation (CSI). CSI is used to treat subclinical microscopic disease (15–23.4 Gy) or established metastatic disease (≥36 Gy). CSI is then followed by focal irradiation of the primary site to a cumulative total dose in the range of 54 Gy. The radiotherapeutic management of MB involves irradiation of a large volume of tissues and results in an increased risk of long-term illness and morbidities [21]. In fact, these childhood cancer survivors are threefold more likely to develop debilitating chronic health conditions as compared to their siblings [23]. The treatment of ependymoma with radiotherapy is unique for these patients who often present at a very early age. Immediate postoperative radiotherapy is used in children as young as 12 months of age [24].

The aim of this study was to compare IMPT and IMRT treatment plans with respect to target volume, location and age for paediatric medulloblastomas and ependymomas to mimic various clinical scenarios. Information from these plans is evaluated to determine the amount of radiation dose to sub-volumes of specific normal tissues. This comprehensive investigation will assist clinicians in the stratification of patients who would most benefit from IMPT compared to IMRT in the future. As well as informing preferred allocation of limited radiotherapy resources, this patient-specific approach to treatment planning is leading the way for personalised medicine.

## 2. Methods

### 2.1. Patient Data and Target Volume Simulation

**Figure 1:**
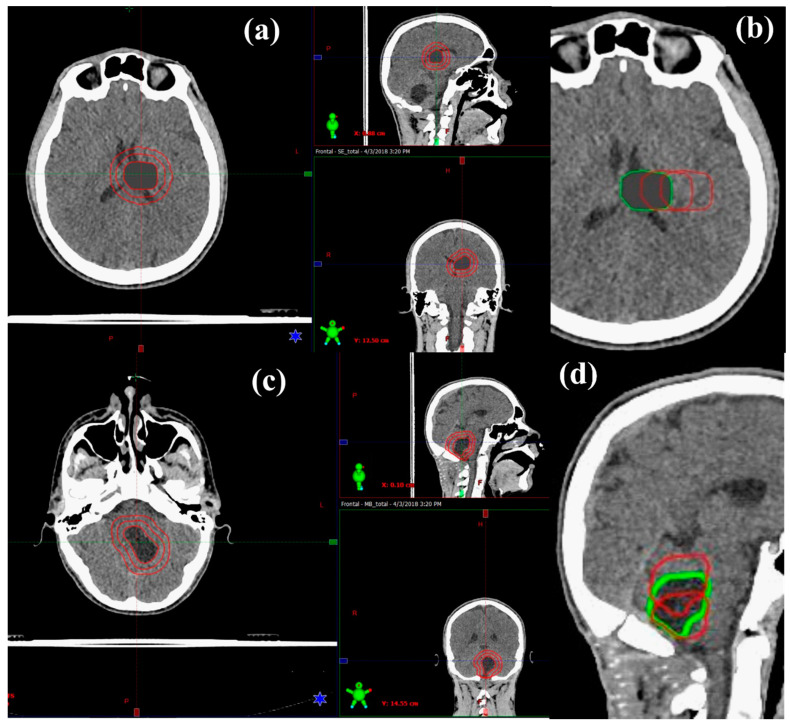
13-year-old male (**a**) Supratentorial ependymoma (STEP) expansions in clinical target volumes in axial, sagittal, and coronal views (from left to right). (**b**) STEP variations in clinical target volume (CTV) location in axial view (green is the original). (**c**) Medulloblastoma (MB) expansions in clinical target volumes in axial, sagittal, and coronal views (from left to right). (**d**) MB variations in CTV location in sagittal view (green is the original).

In this work, CT datasets and respective OAR contours of six paediatric patients who underwent PT with curative intent at St. Jude Children’s Research Hospital were sourced after approvals from relevant ethics committees were obtained from both institutions (St. Jude Children’s Research Hospital and University of South Australia, ethic code: 202267). Six patient datasets were used in total for this modelling study, with two datasets (female and male) for each age group (5, 9 and 13-years-old). These datasets were used as the basis of this study, minimising anatomical variability and allowing for clinical scenarios to be systematically controlled. All critical structures were previously delineated by clinicians. For modelling purposes, in this work, target volumes were added to simulate a range of volumes and shifts corresponding to the anatomical regions of the two tumours, that is infratentorial for MB and supratentorial for ependymoma (see Figure 1). MB and ependymoma were selected based on prevalence and diagnosis, as well as their common anatomic locations within the brain (infratentorial vs. supratentorial) in order to observe the impact of dose to specific normal tissue sub-volumes across the cerebral, IVth ventricle, and cerebellopontine regions. The supratentorial ependymoma (STEP) target volumes were created for each patient to represent an asymmetrical post-surgical tumour-bed volume of approximately 10 mm diameter. The MB plans followed the original clinical target volume (CTV) as a guide (prospectively delineated after surgery for each patient by a radiation oncologist). From this CTV, both target volumes were expanded (or contracted for MB) to create three variations of clinically plausible CTV diameter sizes from 1–3 cm. Similarly, this CTV was shifted, 1 and 2 cm laterally for STEP and 1 cm inferiorly and 1 cm superiorly for MB, to create three CTV locations for each tumour diagnosis. As these tumours may have different locations for different patients, the shifts represent more clinical scenarios and increase the statistical power of the datasets. This resulted in 9 simulated variations for the CTV of STEP and 9 variations for the CTV of MB for each patient. Table 1 describes all CTV variations for STEP and MB, visualised in Figure 1. Additionally, all CTVs were overridden to the density of oedema (11 Hounsfield units) to ensure consistency (post-surgical radiotherapy treatment) across simulated plans. For each IMRT plan, a 3 mm margin was applied to the CTV to create a planning target volume (PTV), whereas for IMPT plans, robust optimisation was performed directly on the CTV as PTV is not commonly used in pencil beam PT optimisation.

### 2.2. Treatment Planning

A total of 216 treatment plans were created, resulting from 18 variations of simulated CTV (9 MB and 9 STEP) and 2 treatment methods for each of the 6 patients. IMPT plans were generated for each simulated CTV following standard clinical planning protocols of two clinical trials SJMB12 (NCT00602667) and SJYC07 (NCT01878617). Similarly, IMRT plans were comparatively created using conventional fractionation. Treatment plans were created by the first author and reviewed by an experienced dosimetrist.

Planning was performed on cranial CT datasets of 1.5 mm slice thickness using Eclipse version 13.7 treatment planning software (Varian Medical Systems, Palo Alto, CA, USA). IMPT plans were optimised using the scenario-based robust optimisation method (3 mm positional and 3% range uncertainty). Both IMRT and IMPT plans met prescription objectives for target volume coverage and were optimised to achieve clinically acceptable OAR doses (see Table 2).

#### 2.2.1. Prescribed Dose and Beam Arrangement for Intensity-Modulated Proton Therapy (IMPT)

All IMPT plans were clinically planned to account for a relative biological effectiveness (RBE) of 1.1 [25] using a generic modulated scanning machine capable of delivering 70–250 MeV beams using a discrete spot-scanning method (spot size, σ~5 mm to 4 mm, in air). A perpendicular two-field arrangement (lateral and posterior) was used to deliver a standard dose prescription of 54 Gy (RBE) in 1.8 Gy (RBE) per fraction for all STEP IMPT plans.

Two posterior-oblique beams were generated to deliver a CSI dose of 23.4 Gy (RBE) to the entire brain in IMPT treatment plans for MB. For 5 and 9-year-old CSI phase plans an additional posterior field was added to improve the coverage of all cranial contents due to the smaller separations of the brain. A primary site boost to 54 Gy (RBE) was then planned to the CTV using two lateral beams with a couch kick angle of 20° to the avoid mastoid air cells and thickest portion of the skull.

#### 2.2.2. Prescribed Dose and Beam Arrangement for Intensity-Modulated Photon Therapy (IMRT)

**Figure 2:**
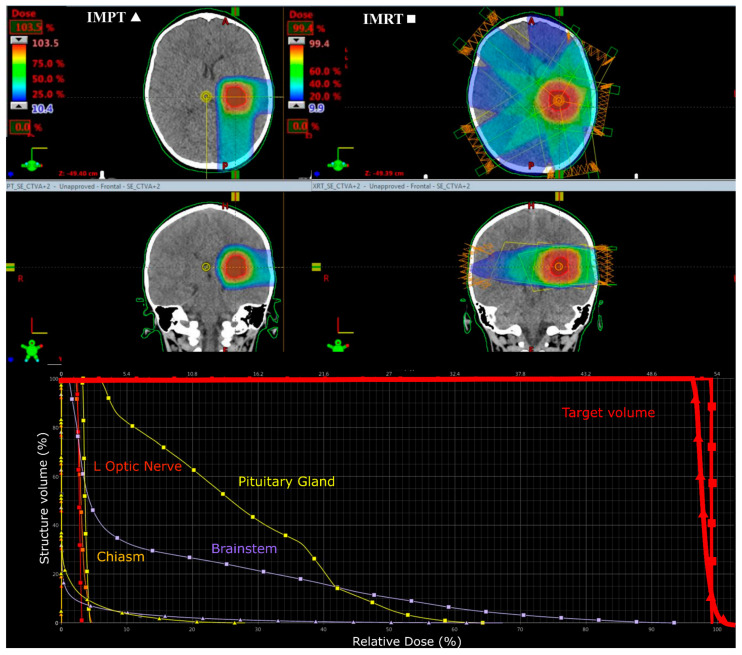
Colourwash dose distributions and dose–volume histograms for a 5-year-old male with supratentorial ependymoma. Typical planning beam arrangements and dose–volume histograms viewed on axial and coronal CT images for intensity-modulated proton therapy (IMPT) (left **▲**) and intensity-modulated photon radiotherapy (IMRT) (right **■**) plans.

**Figure 3:**
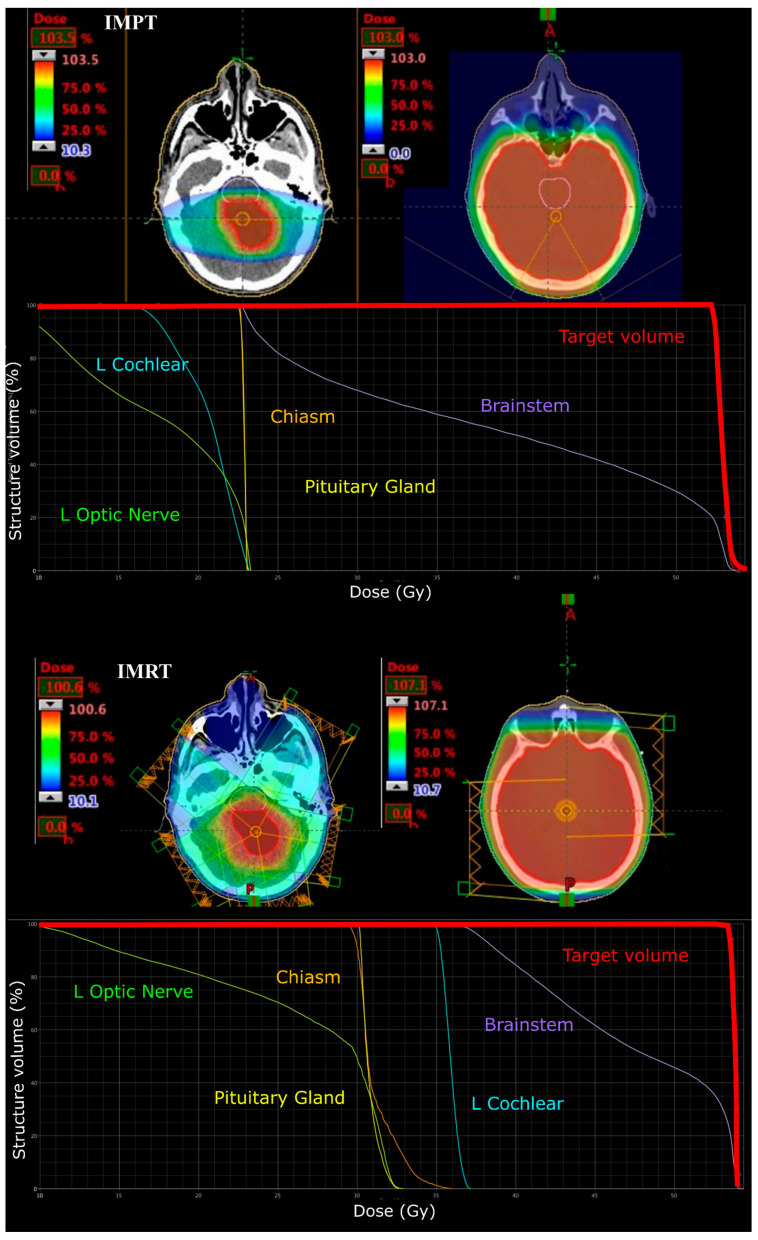
Colourwash dose distributions and dose–volume histograms for a 13-year-old male with infratentorial medulloblastoma. Typical planning beam arrangement viewed on axial CT images for IMPT tumour bed (top left) and craniospinal (top right) and IMRT tumour bed (bottom left) and craniospinal (bottom right) plans. Respective dose–volume histograms are shown below.

All IMRT plans were planned to deliver a prescription of 54 Gy (1.8 Gy per fraction). Five co-planar IMRT photon fields of 6 MV energy were required (gantry angles: 30, 100, 170, 240 and 310 degrees) to adequately cover the target volume (see Figure 2). IMRT plans were optimised to similarly deliver a CSI dose of 24 Gy via opposed lateral fields and a 30 Gy boost phase using a five field co-planar arrangement (see Figure 3).

### 2.3. Dose, Volume, and Statistical Analysis

The respective dose–volume histograms (DVH) were exported from the treatment planning system for dose and volume analysis. For the CTV: maximum, mean, median (volume receiving 50% of the dose (D50)) and minimum as well as the volume receiving at least 95% of the prescription dose (D95) were extracted. Seven critical structures were selected for evaluation based on the proximity to the target and relevance, including the brainstem, optic chiasm, pituitary gland, and ipsilateral cochlea, globe, lens, and optic nerve. The critical structures on the ipsilateral side were evaluated as the lateral shift for STEP target volumes all moved 1 cm or 2 cm laterally (to the left of the patient) and for MB plans the critical structure dose was assumed unilateral as all shifts were along midline. Critical structures for the maximum, mean, and minimum dose were evaluated as well as the volume receiving at least 10% of the dose (D10) were exported. A flow chart of the study design can be found in Figure S1.

Extracted data were transferred to Excel Microsoft spreadsheets (Excel 2010, Microsoft, Redmond, VA, USA) before being uploaded to GraphPad Prism 7 (Version 8, GraphPad Software Inc., Sand Diego, CA, USA). The statistical analysis was performed to assess the impact of target volume size and location across the gender-matched age groups. Paired T-tests were performed to compare the dose and volume data for critical structures between the 108 IMRT and 108 IMPT plans for MB and STEP, respectively. ANOVA testing was performed to investigate the dependence of IMPT on age for mean and D10 dose differences (IMRT-IMPT).

### 2.4. Reliability and Validity

Treatment plans were optimised to achieve the prescriptions while observing OAR dose constraints. To ensure the clinical acceptability as per standard metrics (Table 2) an external paediatric PT planning dosimetrist and experienced XRT planner confirmed the beam placement and dose distributions. Validation of data transfer between Eclipse and Excel was performed by a separate researcher who evaluated 20 randomly selected structures across a 5% sample to ensure data transfer matched the correct target volume/shift in the respective plan.

## 3. Results

All plans were successfully produced, achieving all planning objectives and constraints across all plan pairs as per standard clinical protocols. The following dosimetric results for selected OAR are presented separately for MB and STEP plans.

**Figure 4:**
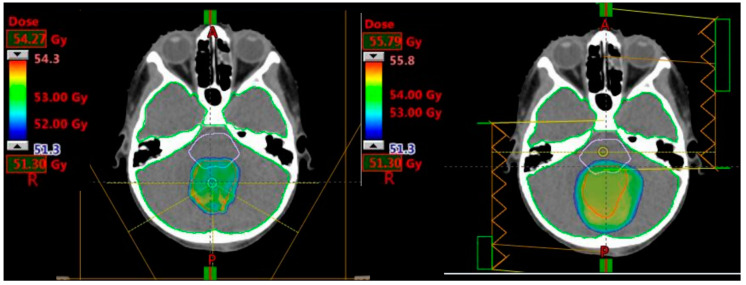
13-year-old female with infratentorial medulloblastoma. Typical composite plan showing D95 coverage of the clinical target volume in IMPT (left) and IMRT (right) plans.

Target volumes achieved ≥95% of the prescribed dose and all OAR were within tolerance levels (Figure 4). There was no significant difference in maximum dose to the CTV averaged across patient plans between modalities (*p* > 0.05), indicating that all plans yielded the same coverage of the CTV.

All IMPT plans across both infratentorial and supratentorial tumour locations demonstrated improved sparing of all critical structures compared to IMRT. The improvements varied significantly across all DVH metrics. Graphical representations of t-test analyses across IMRT and IMPT plans are presented in Figures S2–S12. Table 3 and Table 4 list the average dose difference (∆ IMRT-IMPT) in Gray for mean and D10 of critical structures (across 6 patient plans) collected for STEP and MB cases (both prescribed a total dose of 54 Gy), respectively. For absolute doses, please see Tables S1 and S2. A summary of the overall observations (maximum, median, mean doses, and D10) are shown in Table S3.

Doses to critical structures differed between infratentorial and supratentorial location, since MB plans inherently have higher dose to critical structures than STEP since the intracranial contents were irradiated with the 23.4 Gy/Gy(RBE) CSI phase.

Mean dose and D10 dose difference (IMRT-IMPT) data for critical structures across three patient ages and target shifts (volumes averaged) are summarised in Table S4. Table S4 formed the basis of ANOVA testing to assess the dependence of dose difference (IMRT-IMPT) on age and target shift. Table 5 summarises the significance of interaction factors age and target shift have on OAR dose reduction using IMPT compared to IMRT.

Overall, tumour location and patient age was not associated with significant correlation for dose reduction using IMPT for the ipsilateral (left) cochlea, brainstem and pituitary gland for MB. Patient age showed more dependence for STEP plans as a predictor of the benefits of IMPT, except for ipsilateral cochlea and brainstem. Consequently, STEP and MB results will be reported on separately.

### 3.1. Medulloblastoma

In general, smaller MB target volumes predicted less dosimetric benefit of IMPT for mean and median dose to optical structures (eye, lens, and optic nerve) compared to IMRT across all shifts. The maximum dose to optical structures were similar between modalities, regardless of volume with a superior shift (in general).

As demonstrated in Table 3, the average dose reduction between IMPT and IMRT plans was highest for the brainstem. Figure 5 highlights the dose reduction across maximum, mean and D10 doses whilst all other OAR results can be found in Figures S2–S12.

Large variations in doses to the ipsilateral eye (maximum dose 12–33 Gy) and ipsilateral lens (maximum dose 3–12 Gy) were seen for IMRT plans. All IMRT plans showed a higher dose trend to the ipsilateral eye except the superior shift (contracted target volume) surprisingly did not (median dose for 13-year-old female was 4.1 Gy across both IMRT and IMPT plans). This may have been because the target volume was too small for the lateral shifts to impact the dosimetry between modalities. This was supported by the smallest range of dose to the pituitary gland. The only exception was a negative dosimetric advantage to the ipsilateral optic nerve for some MB plans for individual 13-year-old patients (although minimal 0.1–0.2 Gy). However, the average mean dose to the ipsilateral optic nerve still yielded a positive advantage for IMPT (Table S3).

A large dose reduction was observed for optical structures using IMPT across 5 and 9-year-old patients compared to the 13-year-old. For example, the maximum dose to the ipsilateral optic nerve reduced between IMPT plans by 9 Gy (RBE), 10 Gy (RBE) and 7 Gy (RBE), respectively.

Table 5 demonstrated that the only OAR, which demonstrated a significance between age and location of the target volume (shift) was the optic chiasm (across both mean dose and D10). This is anticipated, as the CTV moved superior, the dose expectedly increased compared to an inferior shift. Across all ages and target volumes, the 1 cm superior shift expectedly increased the average dose difference to the structure for D10 and mean dose (1.5–2 Gy/Gy(RBE)) compared to the central location, demonstrating that paediatric patients of any age with a more superior target volume would benefit from IMPT.

### 3.2. Supratentorial Ependymoma

Similar to infratentorial plans, STEP IMPT plans demonstrated greater dose reductions for OAR. Expectedly, the largest STEP target volume (3 cm diameter), had the most pronounced dose reduction with IMPT across all patients.

**Figure 6:**
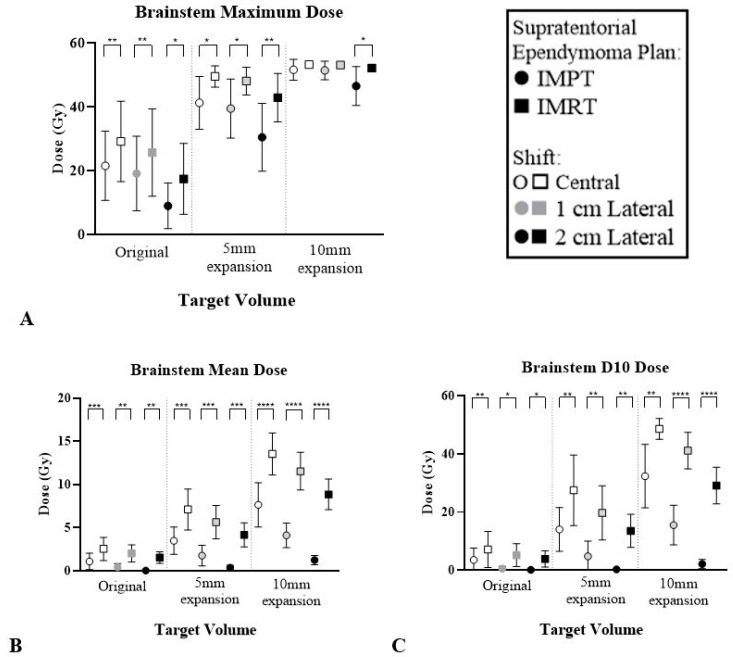
Average dose to brainstem across six patients across IMPT and IMRT plans for three STEP volumes and locations. (**A**) Maximum dose. (**B**) Mean dose. (**C**) Dose to 10% of the structure. Paired *t*-test error bars represent the 95% confidence interval of the mean. * *p* < 0.05, ** *p* < 0.01, *** *p* < 0.001, **** *p* < 0.0001.

**Figure 7:**
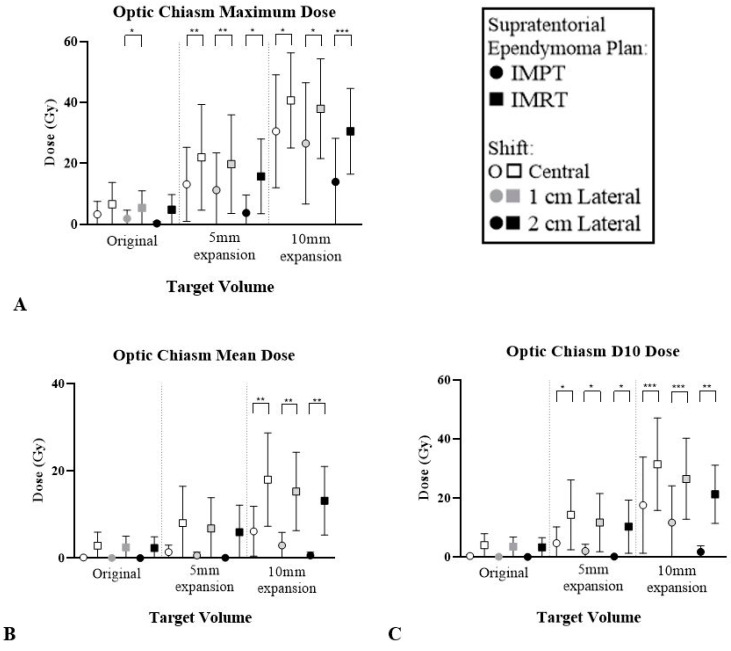
Average dose optic chiasm across six patients across IMPT and IMRT plans for three STEP volumes and locations. (**A**) Maximum dose. (**B**) Mean dose. (**C**) Dose to 10% of the structure. Paired *t*-test error bars represent the 95% confidence interval of the mean. * *p* < 0.05, ** *p* < 0.01, *** *p* < 0.001.

The brainstem and optic chiasm had the most significant reduction with IMPT for STEP. The average dose reduction between modalities was higher for these structures, as demonstrated in Table 3. Figure 6 and Figure 7 highlight the dose reduction across DVH metrics whilst all other OAR results can be found in Figures S8–S12.

Large range of values were observed for optic structures across IMRT plans (i.e., ipsilateral eye maximum dose ranged from 1.4 Gy (9-year-old female) up to 22.7 Gy (13-year-old male) for the same CTV. Most IMRT plans showed a higher mean dose trend across optic structures for the expanded STEP target volumes, increasing exponentially as the target shifted laterally. Similar to MB plans, the target volume reached a size where lateral shifts of the target volume did not reduce the irradiation of evaluated OAR drastically between modalities. Therefore, the larger the STEP target volume (3 cm diameter), the less significant irradiation to the brainstem (maximum) and optic chiasm (mean and median) across lateral shifts (IMPT compared to IMRT).

Larger STEP target volume was associated with a larger mean and D10 dose reduction between modalities for all critical structures (Table S3). IMPT demonstrated dosimetric benefits despite changes in STEP volume and/or laterality increase across the variation of paediatric patient ages. In general, a larger lateral shift (up to 2 cm) was associated with a larger dose reduction using IMPT for critical structures across all target volumes. This was because as the target volume moved away from the central location, the dose was observed to decrease much more with IMPT than IMRT. However, dose to relatively small critical structures such as the cochlea and pituitary gland seemed unchanged between modalities when the target volume was shifted laterally for each of the expansions. Therefore, only the STEP target volume rather than the tumour laterality would impact the degree of dose reduction to cochlea and pituitary gland.

Table 5 demonstrated that age was a strong predictor of dosimetric reduction to the pituitary gland and optical structures. Both the 13-year-old female and male demonstrated significantly larger reductions of dose to the optical structures and pituitary gland across all anatomical shifts using IMPT compared to any other age group. However, there was no correlation between shifts for the STEP.

## 4. Discussion

Gender-matched patients of ages 5, 9, and 13 years old were planned for IMRT and IMPT and compared under a range of simulated scenarios. Seven respective critical structures were evaluated for each of the 216 plans. Despite variations in simulated target volumes and positions, all critical structures were observed to receive lower radiation doses with IMPT plans compared to IMRT. This finding was consistent across all ages and simulated diagnoses: MB and STEP CTVs, demonstrating a benefit from IMPT for patients of all ages, target volumes, and locations. However, Table 5 indicates dosimetric reduction for each critical structure was not equivalent across all patient ages.

Pituitary gland sparing from IMPT was significantly impacted by patient age for those with supratentorial tumours volumes (*p* < 0.001). This reduction in mean dose to the hypothalamic and pituitary region has the clinical potential to reduce side effects such as growth and endocrine deficiency [26,27]. When using fractionated irradiation and the doses planned for children with brain tumours, the hypothalamus is the effector organ for radiation damage and not the pituitary. Because the pituitary is more readily identifiable by most members of the planning team and part of the hypothalamic-pituitary unit, it serves as a reliable surrogate for the hypothalamus.

Only a single medulloblastoma plan (13-year-old female) received a higher D10 brainstem dose in IMPT than IMRT of 0.7 Gy for 1.5 cm diameter target volume in central location (as seen in Table 4). Similarly, this patient showed slightly higher IMPT doses for lens D10 and optic nerve mean dose (0.1 to 0.4 Gy(RBE)) compared to IMRT. This is seen across all shifts for the smaller target volume (0.5 cm diameter), possibly due to the CSI phase or an increased placement of pencil beam spots in this region during the boost phase.

Generally, the superior MB target volumes produced larger dose differences, but the anatomy of a larger skull size was also observed as a contributing factor to the variation in dose reduction across age groups indicating that younger patients may benefit from the dose sparing nature of IMPT. However, based on Rollins, et al. [28] USA circumference growth reference chart, the 13-year-old male head was slightly smaller than average (Table 6).

The 13-year-old male patient received the highest doses to structures across all OAR potentially skewing results for SE plans. Apart from the 13-year-old male, most patients’ average head circumference was similar to the average USA standard (Table 6), except for 5-year-old patients who were slightly smaller. The 13-year-old male measured circumference was in the lower percentile, which could have resulted in increased optic and pituitary dose in STEP plans. The smaller the brain, the closer the critical structures. However, this patient’s head appears to be tilted upwards compared to all other patients performed CT scans (Figure S13). As previously mentioned, the beam arrangements were the same across all patient ages for each of the IMPT and IMRT plans. The optimal beam arrangement could differ from the normal arrangement for the 13-year-old male for STEP. In order to minimise variables, this was not investigated. The 5-year-old patients were anesthetised, which may have prevented tilting, demonstrating that position during CT simulation is also important in helping reduce doses to OARs. Patients treated with conventional CSI often have their head extended to serve at least two purposes. The first, allowing the upper border of the spine field to be as high as possible so that the junction of the cranial fields is not impacted by the shoulders, and the second, to reduce exit dose from the spinal field to the mandible.

Although all results showed that IMPT reduced irradiation to critical structures compared to IMRT across all STEP plans, the estimated dosimetric benefit varied significantly. For example, the cochlea is a smaller critical structure, even though significantly reduced across all DVH metrics the dose was not reduced by a clinically meaningful amount (i.e., IMRT plans irradiated the structure ≤ 2 Gy and the structure was completely spared in IMPT plans). Therefore, the results of this study should be interpreted with a clinical perspective in mind, although any dose reductions are ideal, the degree to which these translate to a clinically meaningful benefit may be limited. In a recent paper, Kahalley, et al. [29] compared PT and XRT treatment in medulloblastoma patients suggesting a meaningful neurocognitive benefit. This reinforces the significance of comparative planning and estimation of NTCP reduction in a clinical setting. Similarly, our findings showed minimal dose, as expected, to the ipsilateral eye, lens, and optic nerve across all IMPT STEP plans (Figure S14). IMPT plans provided dosimetric advantage for the pituitary gland, however, only for larger targets.

An inherent limitation of a retrospective dosimetric study is the inability to assess the impact of confounding variables and lack of recruiter control compared to prospectively designed studies. Additional surgical, chemotherapy and trial information were unavailable for the deidentified data. Additionally, a single planner performed all dosimetric studies and even though reliability and validity were tested through inter- and intra-observer testing, this is still a recognised limitation. Finally, future studies should investigate children under the age of 5-years-old as target volume expansions would help capture the anatomical changes between age groups. Dosimetric planning can be subjective, and clinical practice across institutions will vary.

Standard planning objectives were used across IMRT and IMPT plans to maintain consistency, however, minor variations exist to achieve comparable clinical plan dosimetry for each individual. Beam angles and energy were consistent, however, the manner at which the optimiser works was a case-by-case scenario. Treatment plans for both IMRT and IMPT were clinically acceptable achieving dose objectives for minimum and maximum coverage for each CTV. The 108 IMRT plans optimised slightly higher doses than IMPT, as the algorithm pushed the IMRT plans harder to achieve homogenous dose distributions. The same IMPT dose was received by pituitary gland (maximum, median, and mean dose), ipsilateral optic nerve (all DVH metrics) and optic chiasm (maximum and mean) across all MB IMPT plans demonstrating this consistency.

DVH metrics were chosen based on the frequency of use in the literature, not capturing, however, the full dosimetry and considerations in clinical scenarios. For example, maximum dose could just be a ‘point dose’ in a voxel, while low radiation doses over larger volumes also have detrimental effects. Hence, we attempted to capture this evaluating D10 [30].

### 4.1. Study Significance

To the authors’ knowledge, this is the largest comprehensive paediatric intracranial comparative planning study to date investigating the impact volume and location has on normal tissues across supratentorial and infratentorial tumour sites. All previous MB planning studies retrospectively compared ≤40 plans [31]. Previously, Harrabi, et al. [32] reported the largest retrospective comparative planning study for low-grade glioma (74 patients). However, their patient cohort included patients ranging in age from 2–64 years and compared 3D conformal radiotherapy with pencil beam PT.

As previously reported by our group, most clinical studies compare IMRT and IMPT in terms of patient outcomes. Large scale MB planning studies by Eaton, et al. [33] and Yock, et al. [34] evaluated NTCP and clinical outcomes but provided no DVH analysis. Similarly, Indelicato, et al. [35] and Sato, et al. [22] reported on disease control comparing IMPT and IMRT for 117 and 79 paediatric ependymoma patients with minimal DVH analysis of OAR.

Other groups previously performed comparative planning studies focused on evaluation of critical structures but for different diagnoses [32,36,37,38]. These retrospectively planned studies do not consider similar tumour volume and location as a function of age/gender profile.

As the age of the patient changes, so does the anatomy, in turn influencing the benefits and risks for PT. Proximity of the tumour to OAR changes the across age groups and is, therefore, age-dependent. Merchant, et al. [7] previously linked this to reduced intellectual scores for MB patients. In this study, critical structures were spared from radiation exposure in IMPT plans when OARs were closer to the target volume. The brainstem sparing was consistent across all ages using IMPT, and therefore had no dependence across investigated volumes and locations.

Toussaint, et al. [30] recently performed a comparative planning study to assess the impact of location (posterior fossa ependymoma, craniopharyngioma, and hemispheric ependymoma) dose difference across several brain substructures. However, they found large patient inter-variability across their thirty patients as they were not matched in age or gender. Additionally, the tumours (10 patients per diagnosis) varied in size and were difficult to sort by volume. Brodin, et al. [39] similarly expanded CTV margins to investigate the relationship between target sizes and hippocampal sparing. These were controlled variables in this study, as multiple volumes and locations of the brain were contoured on six patients of three ages rather than assessing a large cohort of paediatric cranial datasets, representing a variation in anatomical target volumes and locations, whilst minimising several anatomical variables that may impact IMRT and IMPT dosimetry. The creation of simulated target volumes for research purposes enabled the investigation of several more relationships between organ at risk sparing and volume/location of targets whilst controlling confounding patient variables, thus reducing the variability that would exist if the plans were created for different patients clinically.

### 4.2. Future Considerations

This study systematically delineated the dosimetric impact of tumour volume, shift, and location on critical structures of paediatric intracranial cancer. Previously published comparative studies evaluating 3D conformal XRT planning techniques for STEP and MB are heavily outdated by the introduction of IMRT and VMAT. MacDonald, et al. [40] published the early clinical outcomes in 2008 for patients undergoing 3D conformal PT for STEP. The group also generated IMRT and IMPT comparative plans to demonstrate improved sparing of the temporal lobes and critical structures such as the brainstem, cochlea, and hypothalamus. These findings support that reduced radiation dose to surrounding critical structures reduces side effects and improves patient outcomes. For example, mean dose received by the pituitary and hypothalamus have previously been linked to reduced cortisol levels and endocrine deficiencies including growth, gonadotropin, and thyroid hormones [26]. Updated clinical trial outcomes for paediatric patients undergoing IMPT are required to investigate whether dosimetric advantages translate to clinical benefits.

We did not require a large number of CT datasets, as we drew various tumour volumes, locations, and even densities (in Hounsfield units) within a CT dataset to simulate various scenarios. As a result, we only required limited CT datasets of varying sizes (i.e., head CT datasets corresponding to different ages or datasets for small, medium and large size patients of both sexes) to use as simulation “phantoms”. In case of this study, these tumours have no pathological or histological characteristics other than location, size, and density, so therefore, the same data would be valid for other cancers in similar locations, which patients would similarly benefit from.

This study assumed the RBE of IMPT plans as ~1.1, although RBE remains an average across tissues [25]. Common IMPT fractionation is 1.8 Gy(RBE) but if the RBE was ≥2 this would drastically change the NTCP effect for critical structures. Giantsoudi, et al. [41] reviewed RBE and LET values for up to 111 paediatric MB patients post-PT and found brainstem injury incidence is equivalent to that of XRT. Additional investigations should take into account the variations of RBE and model the impact on NTCP for all OAR.

## 5. Conclusions

This comprehensive evaluation is the first retrospective study to compare clinically applicable IMPT and IMPT techniques to evaluate the impact tumour volume and locations have on treatment for MB and STEP across three age groups of paediatric patients. This study estimated the benefit of IMPT depending on target volume and/or location as well as the age of the paediatric patient. IMPT demonstrated a dose reduction to normal tissues across a large range of simulated clinical scenarios, particularly significant for centralised tumours, demonstrating a larger dose reduction to optical structures (and pituitary gland *p* < 0.001 in STEP) for 5 and 9-year-old patients compared to older cohorts (13-year-old). However, brainstem sparing was equivalent and unchanged and had no dependence on age across investigated volumes and locations. Critical structure sparing from IMPT was more significantly dependent on age for supratentorial target volumes than infratentorial across a variation of simulated shifts and expansions. Leading the way for personalised medicine, these results will inform a preferred allocation of limited radiotherapy resources and provide a patient-specific treatment approach.

## Figures and Tables

**Figure 5:**
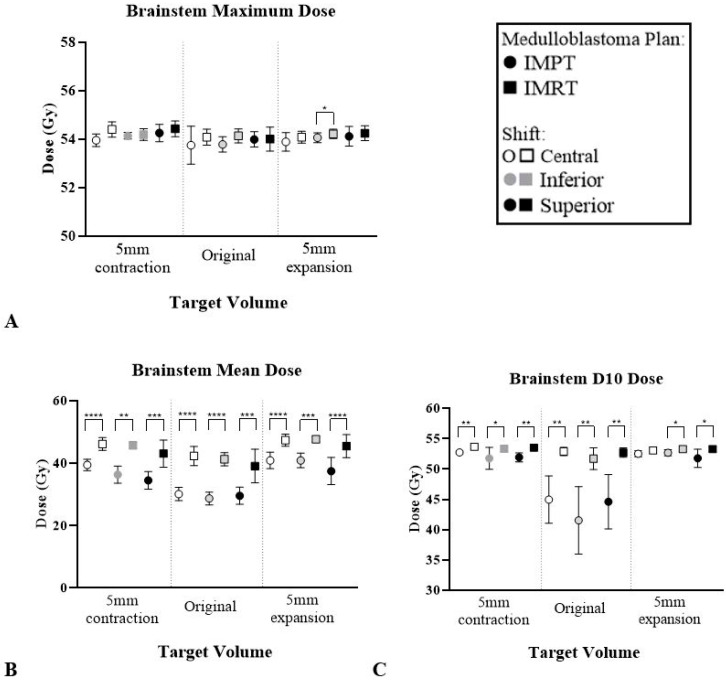
Average dose to brainstem across six patients across IMPT and IMRT plans for three MB volumes and locations. (**A**) Maximum dose. (**B**) Mean dose. (**C**) Dose to 10% of the structure. Paired *t*-test error bars represent the 95% confidence interval of the mean. * *p* < 0.05, ** *p* < 0.01, *** *p* < 0.001, **** *p* < 0.0001.

**Table 1:** Variations in clinical target volume location and size for each tumour diagnosis.

Tumour Site	CTV Location	CTV Size
Infratentorial Medulloblastoma	Close to midline	Original post-surgical target
		−5 mm
		+5 mm
	1 cm superior shift	Original post-surgical target
		−5 mm
		+5 mm
	1 cm inferior shift	Original post-surgical target
		−5 mm
		+5 mm
Supratentorial Ependymoma	Close to midline	15 mm diameter post-surgical target
		+5 mm
		+10 mm
	1 cm lateral shift	15 mm diameter post-surgical target
		+5 mm
		+10 mm
	2 cm lateral shift	15 mm diameter post-surgical target
		+5 mm
		+10 mm

**Abbreviations:** CTV: clinical target volume; +: margin expansion from post-surgical target; −: margin contraction from post-surgical target.

**Table 2:** Dose objectives used for plan evaluation.

Region of Interest	Dosimetric Goals
CTV	D95 ≥ 95%
PTV (IMRT plans)	D90 ≥ 98%
Cochlea	D50 < 20 Gy
Optic Globes	D10 < 35 Gy
	D50 < 10 Gy
Optic Chiasm/Nerves	D10 < 56 Gy
	D50 < 54 Gy
Brainstem	D10 < 52.9 Gy
	D50 < 52.4 Gy
	D0.1 < 53.4 Gy

Abbreviations: CTV: clinical target volume; PTV: planning target volume; IMRT: intensity-modulated radiotherapy.

**Table 3:** Comparison of mean and D10 dose difference (∆ IMRT (Gy)‒IMPT (Gy(RBE))) for critical structures averaged across 6 patient plans collected for supratentorial ependymoma (prescribed dose 54 Gy/Gy(RBE)).

Structure	Dose Parameter (Gy)	1 cm Diameter			2 cm Diameter			3 cm Diameter		
		Central	1 cm LAT	2 cm LAT	Central	1 cm LAT	2 cm LAT	Central	1 cm LAT	2 cm LAT
Brainstem	∆ Mean	1.4 (1.0–2.1) ***	1.5 (1.0–2.7) **	1.5 (0.9–2.7) **	3.6 (2.1–4.8) ***	3.8 (2.2–4.9) ***	3.8 (2.3–5.7) ***	5.8 (4.1–6.6) ****	7.4 (6.0–8.9) ****	7.6 (5.9–9.8) ****
	∆ D10	3.6 (2.1–7.8) **	4.6 (2.4–10.9) *	3.7 (1.9–9.3) *	13.4 (4.5–19.1) **	14.9 (6–20.4) **	13.2 (6.1–22.5) **	16.2 (5.3–26.2) **	25.5 (23.4–28.5) ****	27.0 (21.6–34.8) ****
Optic Chiasm	∆ Mean	2.7 (1.2–8.6)	2.4 (0.9–7.4)	2.3 (0.8–7.4)	6.6 (1.9–21.0)	6.1 (1.7–18.9)	5.8 (1.6–17.8)	11.8 (5.6–22.5) **	12.3 (4.5–22.6) **	12.5 (3.9–24.5) **
	∆ D10	3.6 (1.3–10.8)	3.3 (1.2–9.8)	3.2 (1.1–9.6)	9.5 (2.6–22.2) *	9.6 (2.2–23.7) *	10.1 (2–25.8) *	13.9 (9.3–17.5) ***	14.8 (7.7–18.6) ***	19.5 (6.2–27.9) **
Ipsilateral Cochlea	∆ Mean	0.5 (0.5–0.6) ****	0.5 (0.5–0.6) ****	0.5 (0.5–0.6) ****	0.9 (0.8–0.9) ****	0.9 (0.8–1.0) ****	0.9 (0.8–1.0) ****	1.3 (1.2–1.5) ****	1.4 (1.2–1.5) ****	1.4 (1.2–1.5) ****
	∆ D10	0.5 (0.5–0.6) ****	0.5 (0.5–0.6) ****	0.5 (0.5–0.6) ****	0.9 (0.9–1.0) ****	0.9 (0.9–1.1) ****	0.9 (0.9–1.1) ****	1.4 (1.3–1.6) ****	1.5 (1.3–1.7) ****	1.5 (1.3–1.7) ****
Ipsilateral Eye	∆ Mean	0.9 (0.3–3.6)	0.9 (0.3–3.6)	1.3 (0.3–5.4)	2.1 (0.5–9.1)	1.8 (0.5–6.5)	1.9 (0.6–6.8)	3.8 (0.9–15.4)	3.1 (0.9–10.3)	2.8 (0.9–7.3)
	∆ D10	2.1 (0.4–10.0)	1.5 (0.4–6.5)	1.8 (0.4–7)	3.7 (0.8–17)	2.7 (0.8–8.2)	2.8 (0.8–7.4)	5.7 (1.1–21.9)	5.4 (1.2–19.7)	3.9 (1.2–8.1) *
Ipsilateral Lens	∆ Mean	0.5 (0.3–1.2) *	0.9 (0.3–3.8)	1.3 (0.3–6.0)	1.2 (0.5–4.0)	1.6 (0.5–6.2)	1.8 (0.5–6.4)	3.2 (0.7–13.9)	2.4 (0.8–7.0)	2.6 (0.7–6.7)
	∆ D10	0.5 (0.3–1.5) *	1.2 (0.3–5.5)	1.4 (0.3–6.3)	1.4 (0.5–6.3)	1.7 (0.5–6.4)	2.1 (0.5–6.6)	4.1 (0.8–18.9)	2.8 (0.8–7.5)	2.8 (0.8–6.9)
Ipsilateral Optic Nerve	∆ Mean	1.1 (0.5–3.7)	1.5 (0.5–5.9)	1.6 (0.5–6.7)	3.1 (0.9–13.5)	2.2 (0.9–8.4)	2.2 (0.9–8.2)	4.9 (1.7–20.7)	3.5 (1.7–10.7)	3.1 (1.4–9.6)
	∆ D10	1.5 (0.7–5.5)	1.8 (0.7–7.3)	1.8 (0.7–7.2)	3.9 (1.3–16.8)	2.6 (1.3–9.2)	2.5 (1.2–9)	5.6 (2.2–21.8) *	4.5 (2.0–12.2)	4.1 (1.9–12)
Pituitary Gland	∆ Mean	1.0 (0.7–1.6) ***	0.9 (0.7–1.4) ***	0.8 (0.6–1.3) ***	1.9 (1.3–4.3) **	1.7 (1.2–3.5) **	1.5 (1.0–3.3) **	4.2 (2.0–13.3)	4.1 (1.8–13.7)	3.7 (1.7–12.4)
	∆ D10	1.1 (0.8–1.9) **	1.0 (0.8–1.7) ***	0.8 (0.7–1.5) ***	2.4 (1.4–6.5) *	2.1 (1.3–5.4) *	1.9 (1.2–5.2) *	5.4 (2.3–18.6)	5.3 (2.1–19)	4.7 (1.9–17.2)

**Key:** ∆; dose difference (IMRT-IMPT), D10; dose received by 10% of the structure volume, mean; mean dose received by the structure, central; central target volume, SUP; superior shift of target volume, INF; inferior shift of target volume, LAT; lateral shift of target volume, *; *p* < 0.05, **; *p* < 0.01, ***; *p* < 0.001, ****; *p* < 0.0001.

**Table 4:** Comparison of mean and D10 dose difference (∆ IMRT (Gy)‒IMPT (Gy(RBE))) for critical structures averaged across 6 patient plans collected for infratentorial medulloblastoma (prescribed dose of 54 Gy/Gy(RBE) (23.4 Gy/Gy(RBE) craniospinal irradiation with 30.6 Gy/Gy(RBE) boost)).

Structure	Dose Parameter (Gy)	1 cm Diameter			0.5 cm Diameter			1.5 cm Diameter		
		Central	1 cm SUP	1 cm INF	Central	1 cm SUP	1 cm INF	Central	1 cm SUP	1 cm INF
Brainstem	∆ Mean	6.6 (4.8–8.2) ****	9.3 (5.1–12.1) ***	8.5 (5.3–11.8) ***	12.1 (9.4–15.6) ****	12.5 (10.9–14.3) ****	9.5 (5.6–14.2) ***	6.4 (5.3–8.6) ****	6.8 (4.4–8.9) ***	7.9 (6.3–9.7) ****
	∆ D10	0.9 (0.3–1.5) **	1.5 (0.5–4.3) *	1.4 (0.4–2.3) **	7.9 (3.3–11.8) **	10.1 (5.5–15.8) **	8.0 (1.9–14.2) **	0.5 (−0.7–1.5)	0.6 (0.1–1.6) *	1.5 (0.3–3.5) *
Optic Chiasm	∆ Mean	7.8 (2.4–10.4) **	9.5 (6.9–11.4) ****	6.0 (1.9–9.8) **	6.1 (1.9–8.8) **	7.0 (3.8–8.7) ***	4.1 (1.5–7.7) *	10.5 (3.8–14.8) **	12.6 (9.4–15.1) ****	8.5 (2.1–13.0) **
	∆ D10	9.4 (2.8–12.9) **	11.8 (9.0–13.9) ****	7.5 (2.1–12.7) **	7.5 (2.2–12.6) **	8.5 (4.9–12.3) ***	5.2 (1.5–10.7) *	12.4 (4.9–17.5) ***	14.7 (13–17.7) ****	10.2 (2.4–14.1) **
Ipsilateral Cochlea	∆ Mean	12.7 (10.6–15.2) ****	12.3 (11.0–13.3) ****	12.6 (12.2–13.3) ****	13.3 (11.6–14.4) ****	10.2 (3.8–13.9) **	13.0 (11.7–14.3) ****	12.5 (10.7–15.1) ****	13.1 (10.6–14.6) ****	12.7 (11.8–13.7) ****
	∆ D10	12.1 (8.3–13.9) ****	12.0 (10.9–13.6) ****	12.3 (11.1–13.2) ****	12.8 (11.4–13.7) ****	10.2 (3.9–13.2) **	11.0 (3.1–13.1) **	12.1 (9.8–14.1) ****	12.8 (10.3–14) ****	12.7 (11–13.7) ****
Ipsilateral Eye	∆ Mean	5.2 (2.1–7.3) ***	5.0 (2.3–7.3) **	5.3 (2.0–7.4) ***	3.9 (2.0–5.8) ***	3.5 (1.4–5.6) **	3.8 (1.9–5.6) ***	7.6 (2.2–10.8) **	7.4 (3.1–10.3) **	6.8 (2.1–10.5) **
	∆ D10	7.4 (3.0–10.1) ***	7.4 (3.0–11.5) **	7.3 (2.8–9.5) ***	6.2 (2.8–8.6) ***	5.7 (2.9–9.3) **	6.1 (2.8–8.4) ***	9.7 (3.0–12.9) **	10.0 (3.3–15.4) **	8.9 (2.9–12.3) **
Ipsilateral Lens	∆ Mean	3.4 (1.9–5.3) **	3.1 (0.6–5.1) **	3.5 (2.2–5.0) ***	2.7 (0.2–4.6) **	2.6 (0.0–4.6) **	2.6 (0.0–4.5) **	5.9 (2.3–8.0) **	5.1 (2.6–7.6) **	5.2 (2.2–8.7) **
	∆ D10	4.4 (2.3–7.1) **	3.8 (0.6–6.9) *	5.0 (2.2–6.9) ***	3.3 (–0.1–6.4) *	3.3 (–0.4–6.3) *	3.3 (–0.3–6.2) *	7.2 (2.4–10.4) **	6.3 (2.6–9.7) **	6.6 (2.3–11.1) **
Ipsilateral Optic Nerve	∆ Mean	6.9 (0.0–9.3) **	7.6 (0.9–10.6) **	6.1 (0.0–9.6) **	4.2 (–0.1–6.1) **	4.0 (0.0–5.9) **	3.5 (–0.2–5.3) **	10.5 (0.3–13.6) **	11.5 (3.4–14.9) **	8.5 (0.0–13.3) **
	∆ D10	8.2 (2.2–11.3) **	9.6 (4.2–11.6) ***	7.1 (2.0–22.3) **	5.4 (2.0–8.0) **	5.4 (2.1–7.0) ***	3.9 (1.6–7.5) **	11.2 (2.5–14.6) **	12.3 (7–14.9) ***	9.5 (2–14.4) **
Pituitary Gland	∆ Mean	8.4 (5.2–11.2) ***	9.2 (7.9–11.2) ****	6.9 (1.9–11.6) **	6.5 (2.9–8.8) ***	7.0 (6.7–7.2) ****	5.2 (1.5–7.7) **	11.7 (7.4–14.8) ***	13.1 (11.1–15.4) ****	9.4 (3.2–13.1) **
	∆ D10	9.3 (6.0–12.2) ***	10.6 (9.0–12.8) ****	7.7 (2.0–12.7) **	7.1 (3.8–11.3) ***	7.1 (6.9–7.6) ****	5.4 (1.5–8.9) **	12.7 (8.7–15.2) ****	14.2 (12.4–17.2) ****	10.0 (4.0–13.2) **

**Key:** ∆; dose difference (IMRT-IMPT), D10; dose received by 10% of the structure volume, mean; mean dose received by the structure, central; central target volume, SUP; superior shift of target volume, INF; inferior shift of target volume, LAT; lateral shift of target volume, *; *p* < 0.05, **; *p* < 0.01, ***; *p* < 0.001, ****; *p* < 0.0001.

**Table 5:** Dependence of dose reduction (∆ IMRT-IMPT) on patient age and target shift.

Structure	Dose Parameter (Gy)	Infratentorial Medulloblastoma (*p* Value)				Supratentorial Ependymoma (*p* Value)			
		Age (years)			Shift	Age (years)			Shift
		5 vs. 9	9 vs. 13	5 vs. 13		5 vs. 9	9 vs. 13	5 vs. 13	
Brainstem	∆ Mean	−	−	−	−	−	−	−	−
	∆ D10	−	−	−	−	−	−	−	−
Optic Chiasm	∆ Mean	−	−	−	0.0197 *	−	0.0009	0.0006	−
	∆ D10	−	−	−	0.0231 *	−	−	0.0245	−
Ipsilateral Cochlea	∆ Mean	−	−	−	−	−	−	−	−
	∆ D10	−	−	−	−	−	−	−	−
Ipsilateral Eye	∆ Mean	−	0.0020	0.0172	−	0.0293	0.0164	−	−
	∆ D10	−	0.0002	0.0268	−	0.0257	0.0498	−	−
Ipsilateral Lens	∆ Mean	−	0.0197	0.0063	−	−	0.0019	0.0195	−
	∆ D10	−	0.0095	0.0042	−	−	0.0109	−	−
Ipsilateral Optic Nerve	∆ Mean	−	0.0034	−	−	−	0.0264	0.0367	−
	∆ D10	−	−	−	−	−	0.0264	0.0400	−
Pituitary Gland	∆ Mean	−	−	−	−	0.0010	0.0008	0.0009	−
	∆ D10	−	−	−	−	−	0.0011	0.0009	−

**Key:** ∆; dose difference (IMRT-IMPT), mean; mean dose received by the structure, D10; dose received by 10% of the structure volume, −; no dependence, *; significant *p* value difference for the group (6 patients).

**Table 6:** Comparison of head circumference of current study to report by Rollins, et al. [28] reference chart.

Age and Gender	Circumference—Current Study	Circumference—Referenced Study
5 Female	47.7 cm	50.5 cm
5 Male	49.0 cm	51.5 cm
9 Female	51.5 cm	52.0 cm
9 Male	52.0 cm	53.0 cm
13 Female	53.5 cm	53.5 cm
13 Male	51.0 cm	54.5 cm

## Supplementary Materials

The following are available online at https://www.mdpi.com/2072-6694/12/9/2578/s1, Figure S1: Flowchart of study design, Figure S2: Infratentorial Tumour Location (Medulloblastoma) OAR results- Average dose to optic chiasm across six patients across IMPT and IMRT plans for three MB volumes and locations, Figure S3: Infratentorial Tumour Location (Medulloblastoma) OAR results- Average dose to ipsilateral cochlea across six patients across IMPT and IMRT plans for three MB volumes and locations, Figure S4: Infratentorial Tumour Location (Medulloblastoma) OAR results- Average dose to ipsilateral eye across six patients across IMPT and IMRT plans for three MB volumes and locations, Figure S5: Infratentorial Tumour Location (Medulloblastoma) OAR results- Average dose to ipsilateral lens across six patients across IMPT and IMRT plans for three MB volumes and locations, Figure S6: Infratentorial Tumour Location (Medulloblastoma) OAR results- Average dose to ipsilateral optic nerve across six patients across IMPT and IMRT plans for three MB volumes and locations, Figure S7: Infratentorial Tumour Location (Medulloblastoma) OAR results- Average dose to pituitary gland across six patients across IMPT and IMRT plans for three MB volumes and locations, Figure S8: Supratentorial Tumour Location (Ependymoma) OAR results- Average dose to ipsilateral cochlea across six patients across IMPT and IMRT plans for three STEP volumes and locations, Figure S9: Supratentorial Tumour Location (Ependymoma) OAR results-Average dose to ipsilateral eye across six patients across IMPT and IMRT plans for three STEP volumes and locations, Figure S10: Supratentorial Tumour Location (Ependymoma) OAR results- Average dose to ipsilateral lens across six patients across IMPT and IMRT plans for three STEP volumes and locations, Figure S11: Supratentorial Tumour Location (Ependymoma) OAR results-Average dose to ipsilateral optic nerve across six patients across IMPT and IMRT plans for three STEP volumes and locations, Figure S12: Supratentorial Tumour Location (Ependymoma) OAR results- Average dose to pituitary gland across six patients across IMPT and IMRT plans for three STEP volumes and locations, Figure S13: 13-year-old male head position for CT simulation compared to the rest of the cohort, Figure S14: Ipsilateral eye, lens and optic nerve sparing across IMPT (left) and IMRT (right) plans for STEP clinical volumes in 13-year-old female patient, Table S1: The absolute dose ranges for IMPT (Gy(RBE)) plans (grey shading) and IMRT (Gy) plans (white) for critical structures averaged across 6 patient plans collected for supratentorial ependymoma (prescribed dose 54 Gy/Gy(RBE))**,** Table S2: The absolute dose ranges for IMPT (Gy(RBE)) plans (grey shading) and IMRT (Gy) plans (white) for critical structures averaged across 6 patient plans collected for infratentorial medulloblastoma (prescribed dose of 54 Gy/Gy(RBE) (23.4 Gy/Gy(RBE) craniospinal irradiation with 30.6 Gy/Gy(RBE) boost)), Table S3: Summary of T-test observations comparing significant differences between IMPT and IMRT plans for a range of structures and their respectively collected DVH metrics (maximum, mean, median and D10), Table S4: Mean and D10 dose difference (∆ IMRT (Gy)‒IMPT (Gy(RBE))) for critical structures across 3 patient ages and location (volumes averaged). Prescribed dose for both tumour sites was 54 Gy/Gy(RBE) (23.4 Gy/Gy(RBE) craniospinal irradiation with 30.6 Gy/Gy(RBE) boost for medulloblastoma).
