# Supplementary material for: Influence of Target Location, Size, and Patient Age on Normal Tissue Sparing- Proton and Photon Therapy in Paediatric Brain Tumour Patient-Specific Approach
Source: Cancers (Basel). 2020 Sep 10;12(9):2578. doi: 10.3390/cancers12092578 (PMC7563785; doi:10.3390/cancers12092578)
Supplement: Supplementary file 1 [file cancers-12-02578-s001.pdf]

Supplementary Figure S1. Flowchart of study design.

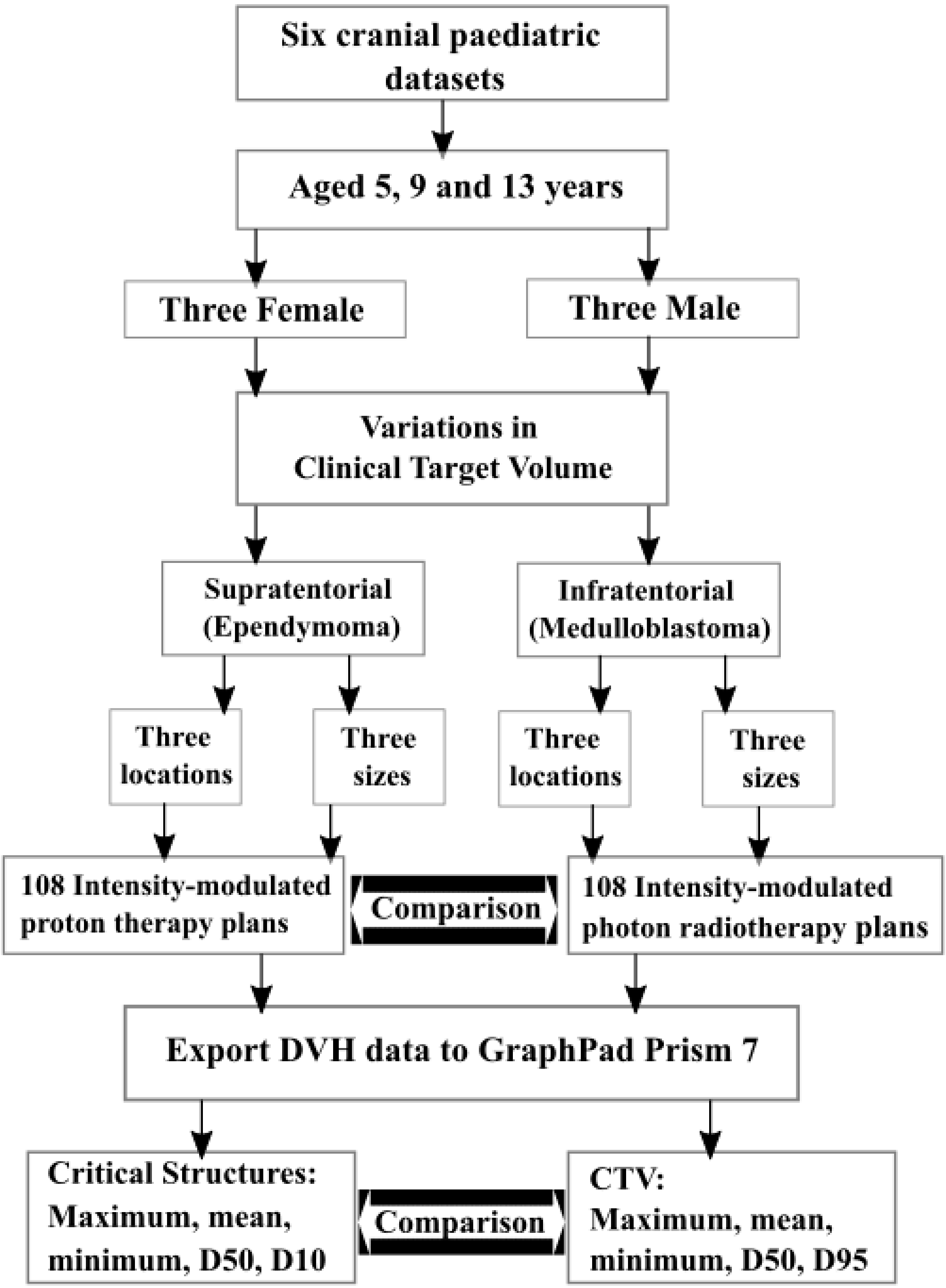

Figure S1. Flowchart of study design.

Supplementary Figures S2–S7. Infratentorial Tumour Location (Medulloblastoma) OAR results.

S2: OPTIC CHIASM RESULTS

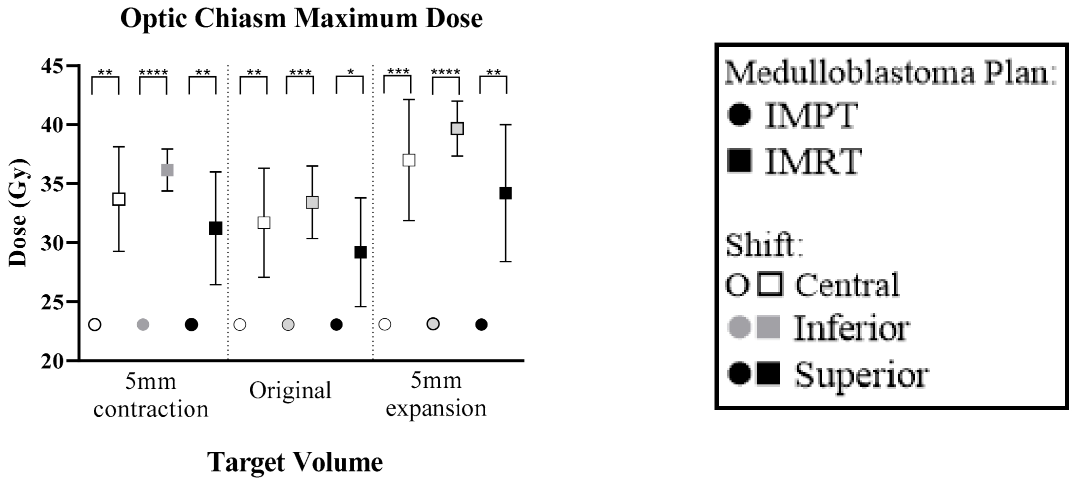

A

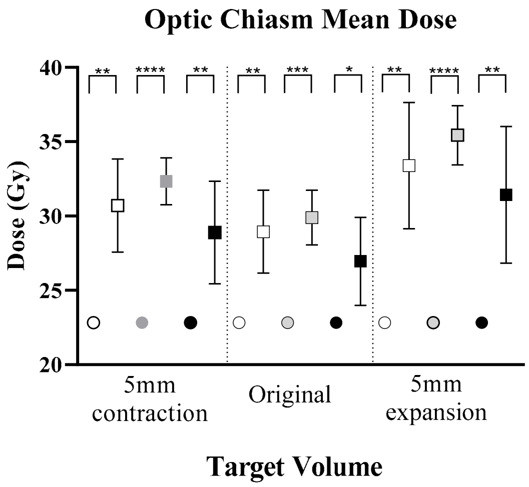

B

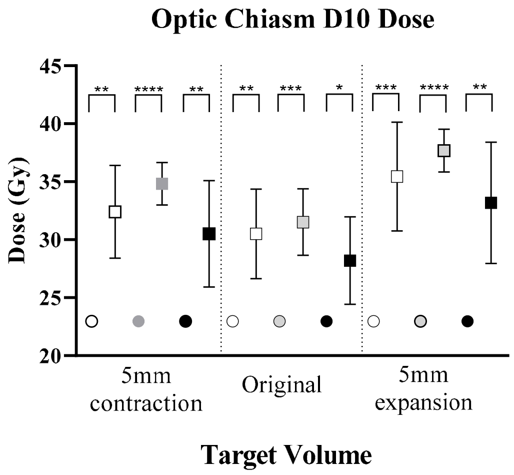

C

Figure S2. Average dose to optic chiasm across six patients across IMPT and IMRT plans for three MB volumes and locations. (**A**) Maximum dose. (**B**) Mean dose. (**C**) Dose to 10% of the structure. Paired T-test error bars represent the 95% confidence interval of the mean. * $p < 0.05$, ** $p < 0.01$, *** $p < 0.001$, **** $p < 0.0001$.

S3: IPSILATERAL (LEFT) COCHLEA RESULTS

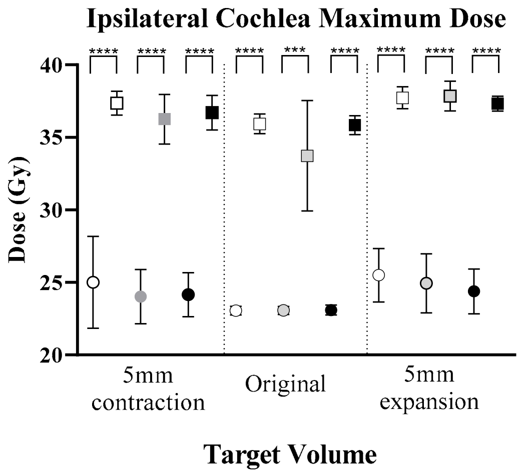

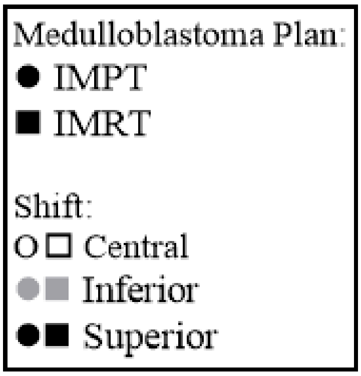

A

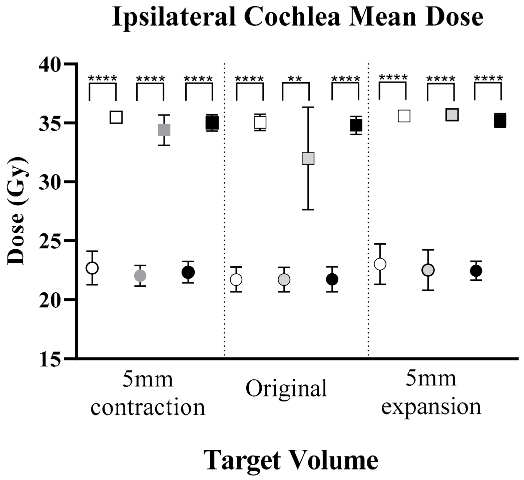

B

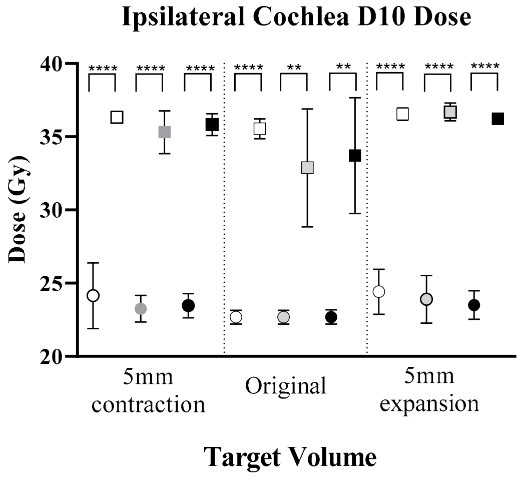

C

Figure S3. Average dose to ipsilateral cochlea across six patients across IMPT and IMRT plans for three MB volumes and locations. (**A**) Maximum dose. (**B**) Mean dose. (**C**) Dose to 10% of the structure. Paired T-test error bars represent the 95% confidence interval of the mean. * $p < 0.05$, ** $p < 0.01$, *** $p < 0.001$, **** $p < 0.0001$.

S4: IPSILATERAL (LEFT) EYE RESULTS

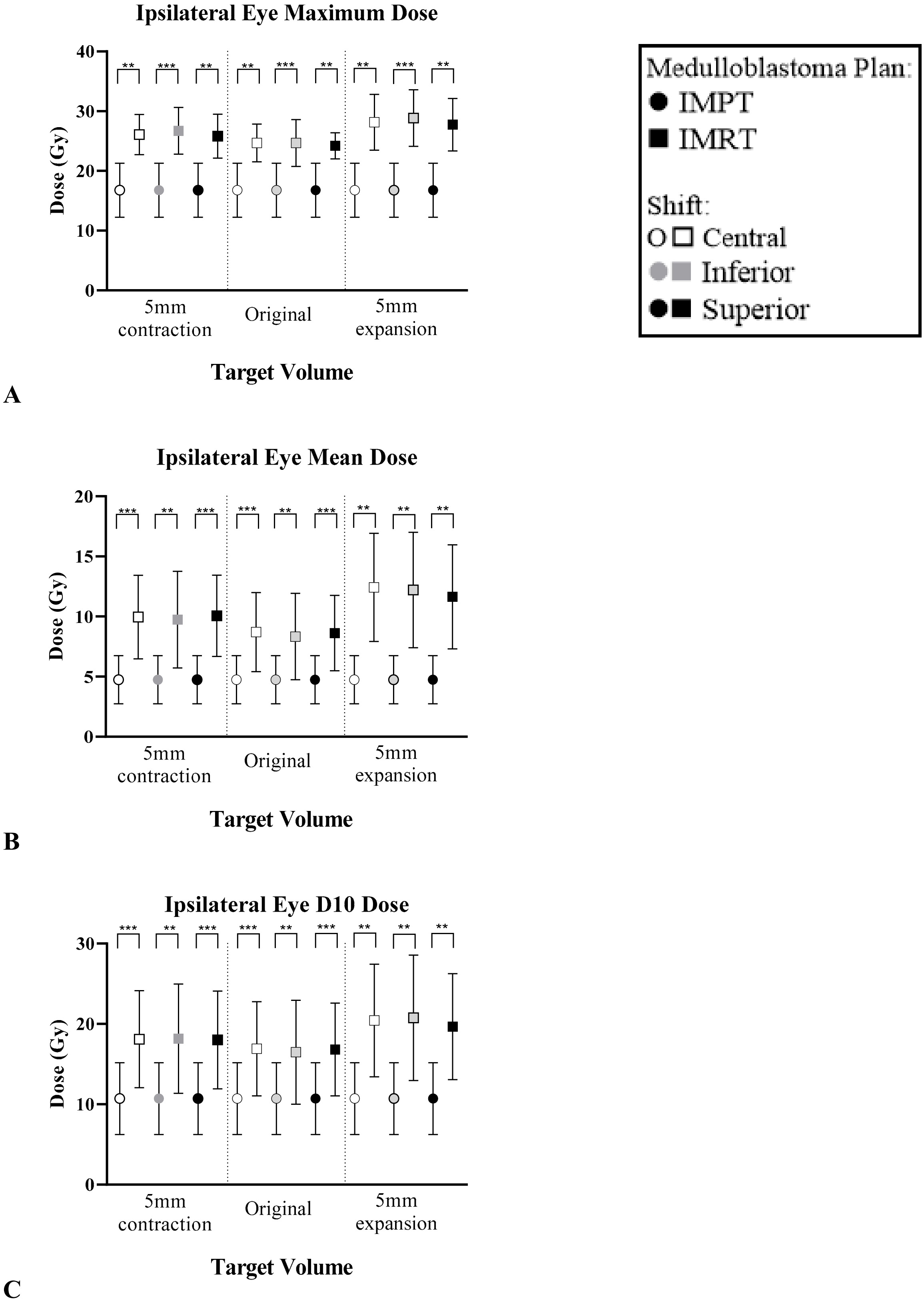

Figure S4. Average dose to ipsilateral eye across six patients across IMPT and IMRT plans for three MB volumes and locations. (**A**) Maximum dose. (**B**) Mean dose. (**C**) Dose to 10% of the structure. Paired T-test error bars represent the 95% confidence interval of the mean. * $p < 0.05$, ** $p < 0.01$, *** $p < 0.001$, **** $p < 0.0001$.

S5: IPSILATERAL (LEFT) LENS RESULTS

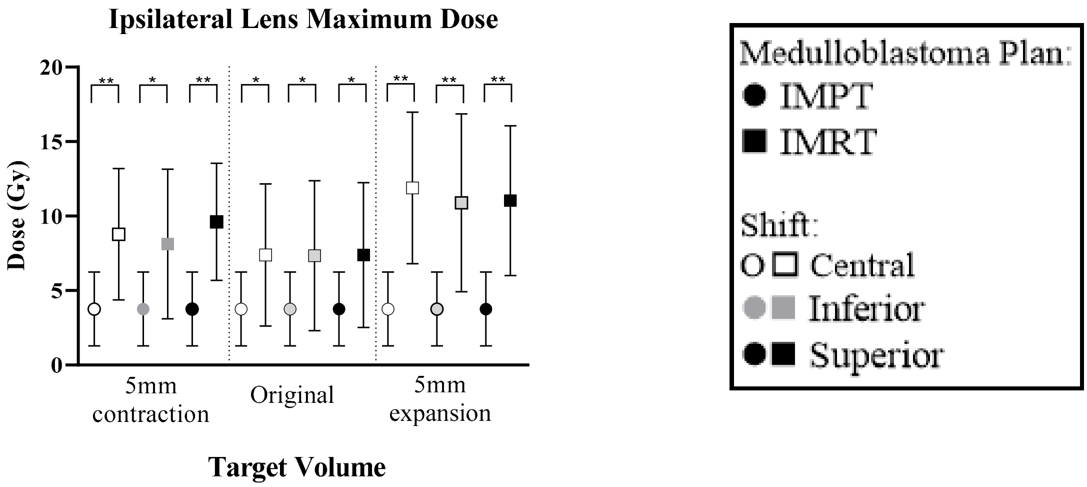

A

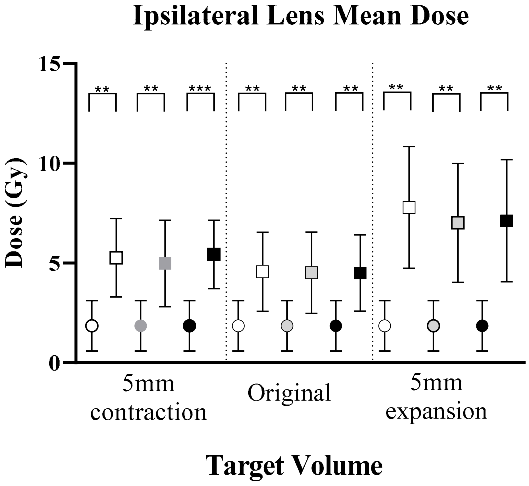

B

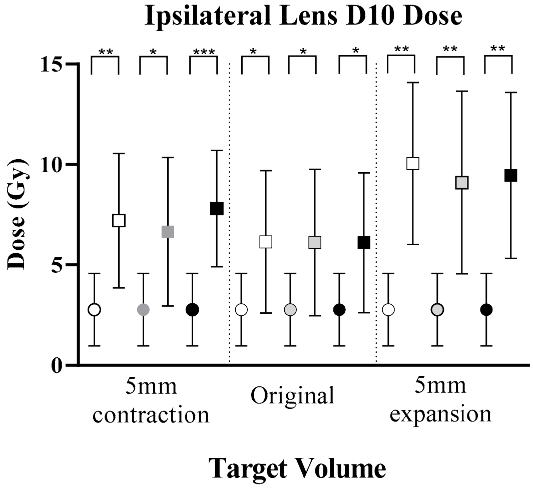

C

Figure S5. Average dose to ipsilateral lens across six patients across IMPT and IMRT plans for three MB volumes and locations. (**A**) Maximum dose. (**B**) Mean dose. (**C**) Dose to 10% of the structure. Paired T-test error bars represent the 95% confidence interval of the mean. * $p < 0.05$, ** $p < 0.01$, *** $p < 0.001$, **** $p < 0.0001$.

S6: IPSILATERAL (LEFT) OPTIC NERVE RESULTS

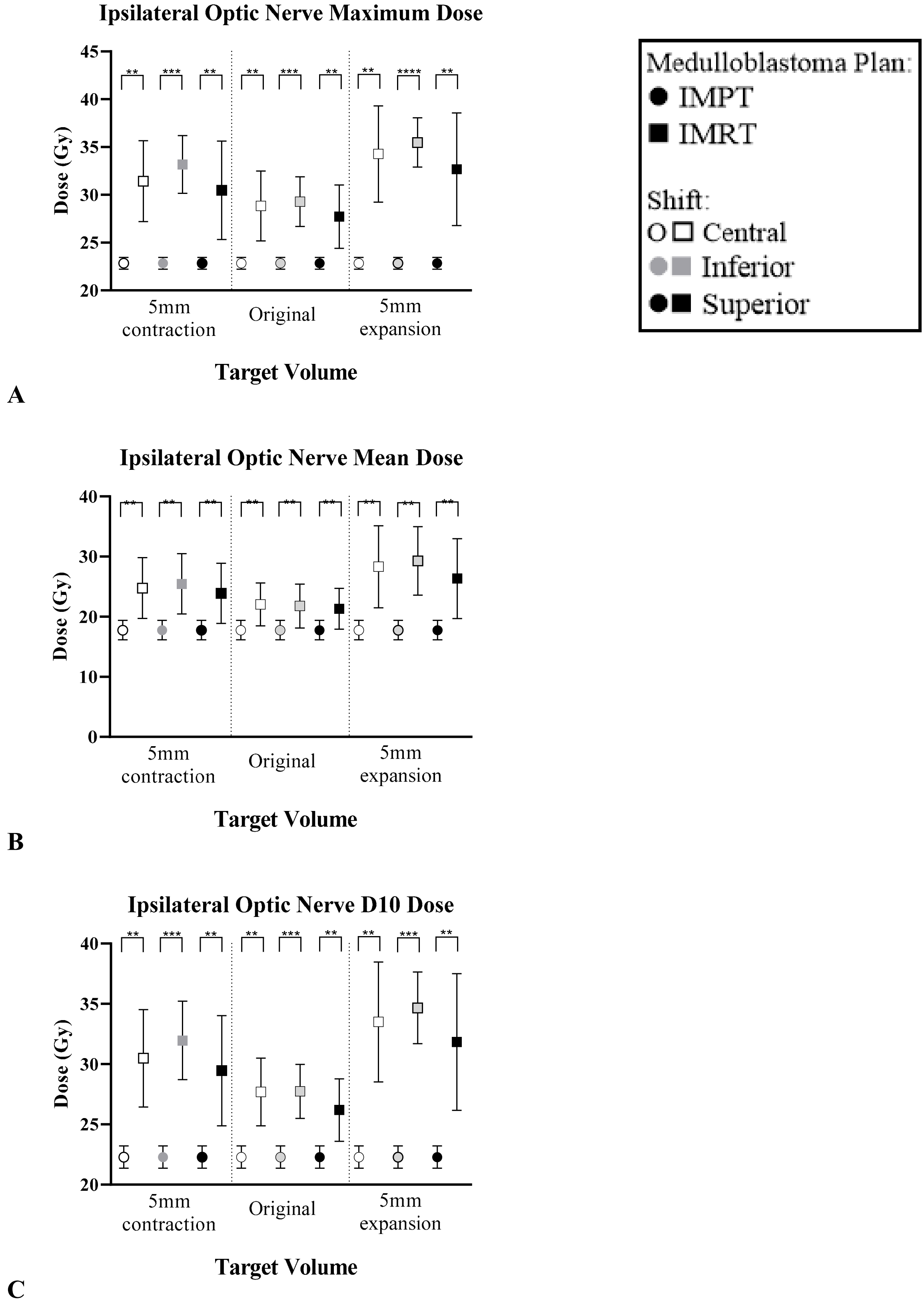

Figure S6. Average dose to ipsilateral optic nerve across six patients across IMPT and IMRT plans for three MB volumes and locations. (**A**) Maximum dose. (**B**) Mean dose. (**C**) Dose to 10% of the structure. Paired T-test error bars represent the 95% confidence interval of the mean. * $p < 0.05$, ** $p < 0.01$, *** $p < 0.001$, **** $p < 0.0001$.

S7: PITUITARY GLAND RESULTS

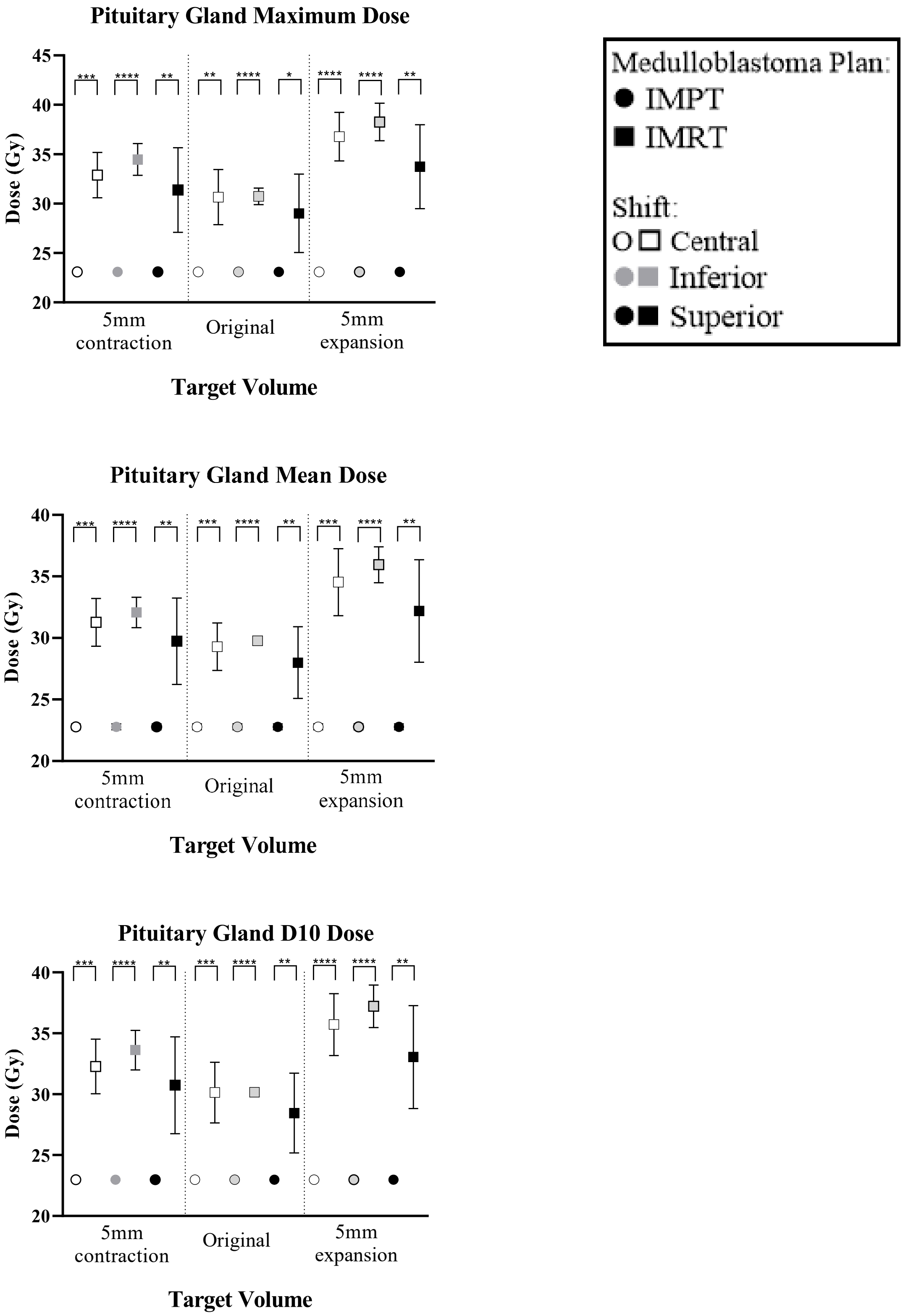

A

B

C

Figure S7. Average dose to pituitary gland across six patients across IMPT and IMRT plans for three MB volumes and locations. (**A**) Maximum dose. (**B**) Mean dose. (**C**) Dose to 10% of the structure. Paired T-test error bars represent the 95% confidence interval of the mean. * $p < 0.05$, ** $p < 0.01$, *** $p < 0.001$, **** $p < 0.0001$.

Supplementary Figures S8–S12. Supratentorial Tumour Location (Ependymoma) OAR results.

S8: IPSILATERAL (LEFT) COCHLEA RESULTS

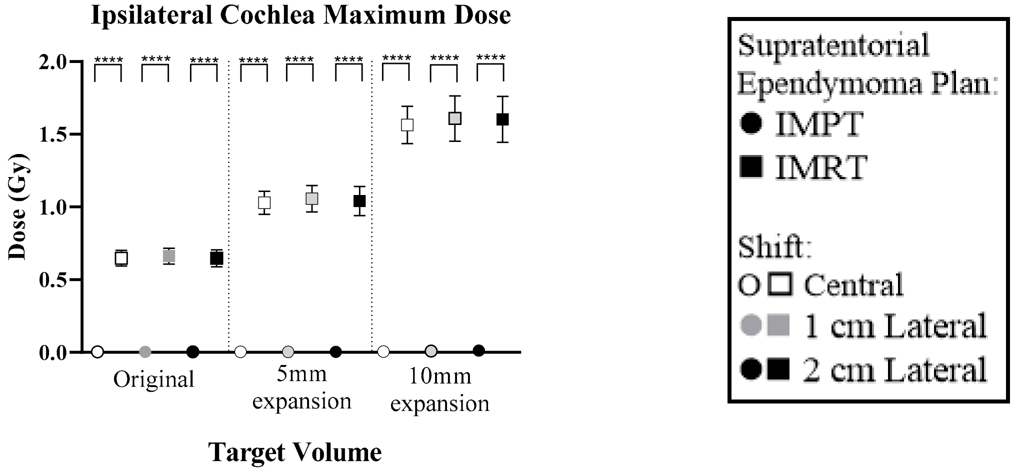

A

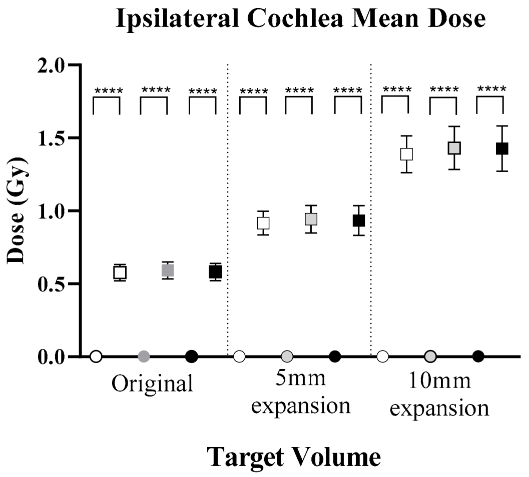

B

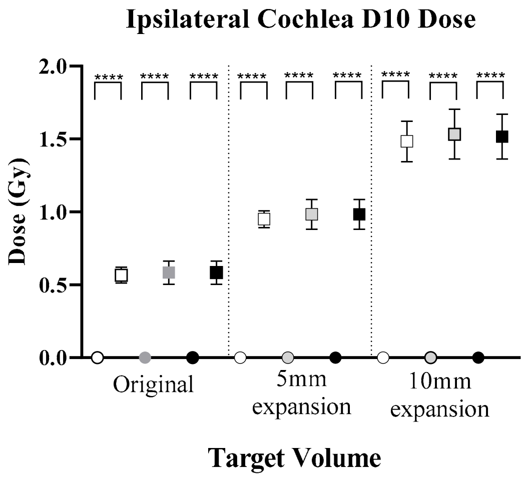

C

Figure S8. Average dose to ipsilateral cochlea across six patients across IMPT and IMRT plans for three STEP volumes and locations. (**A**) Maximum dose. (**B**) Mean dose. (**C**) Dose to 10% of the structure. Paired T-test error bars represent the 95% confidence interval of the mean. * $p < 0.05$, ** $p < 0.01$, *** $p < 0.001$, **** $p < 0.0001$.

S9: IPSILATERAL (LEFT) EYE RESULTS

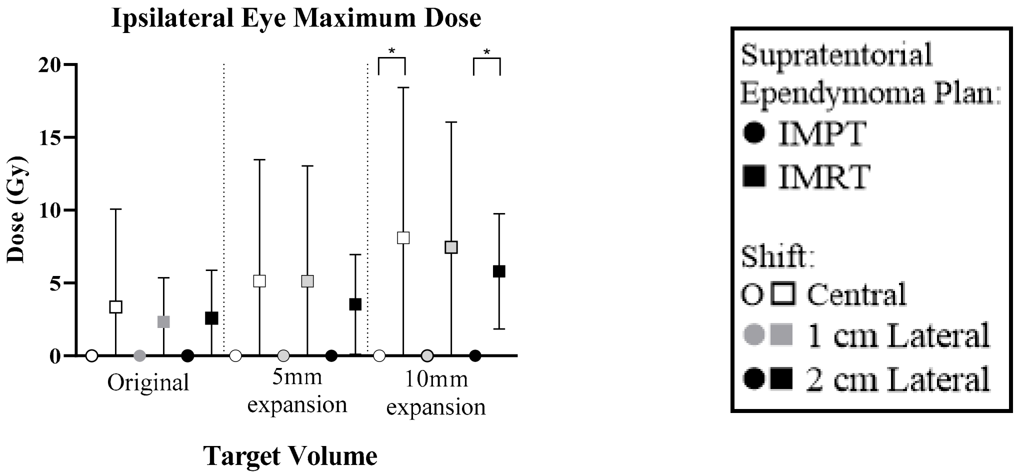

A

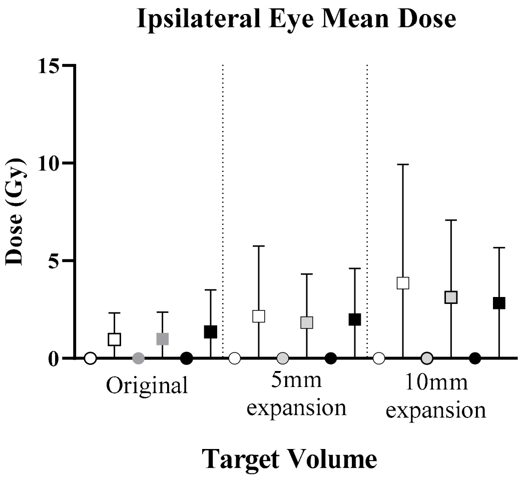

B

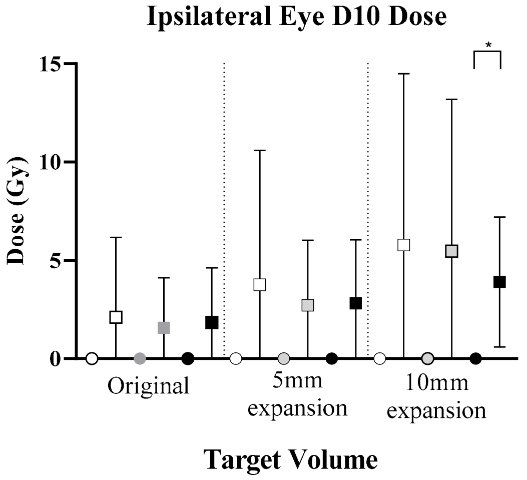

C

Figure S9. Average dose to ipsilateral eye across six patients across IMPT and IMRT plans for three STEP volumes and locations. (**A**) Maximum dose. (**B**) Mean dose. (**C**) Dose to 10% of the structure. Paired T-test error bars represent the 95% confidence interval of the mean. * $p < 0.05$, ** $p < 0.01$, *** $p < 0.001$, **** $p < 0.0001$.

S10: IPSILATERAL (LEFT) LENS RESULTS

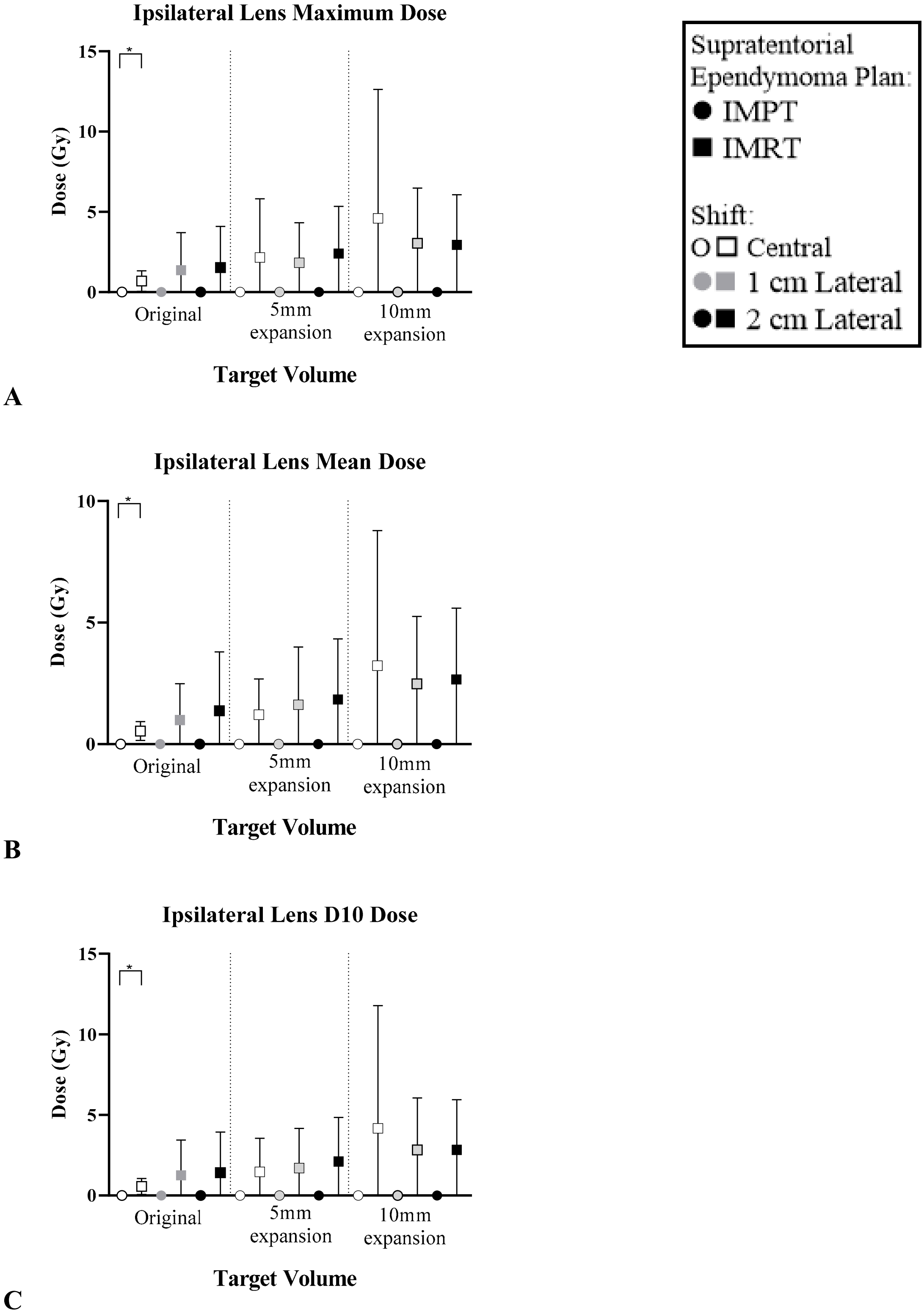

Figure S10. Average dose to ipsilateral lens across six patients across IMPT and IMRT plans for three STEP volumes and locations. (**A**) Maximum dose. (**B**) Mean dose. (**C**) Dose to 10% of the structure. Paired T-test error bars represent the 95% confidence interval of the mean. * $p < 0.05$, ** $p < 0.01$, *** $p < 0.001$, **** $p < 0.0001$.

S11: IPSILATERAL (LEFT) OPTIC NERVE RESULTS

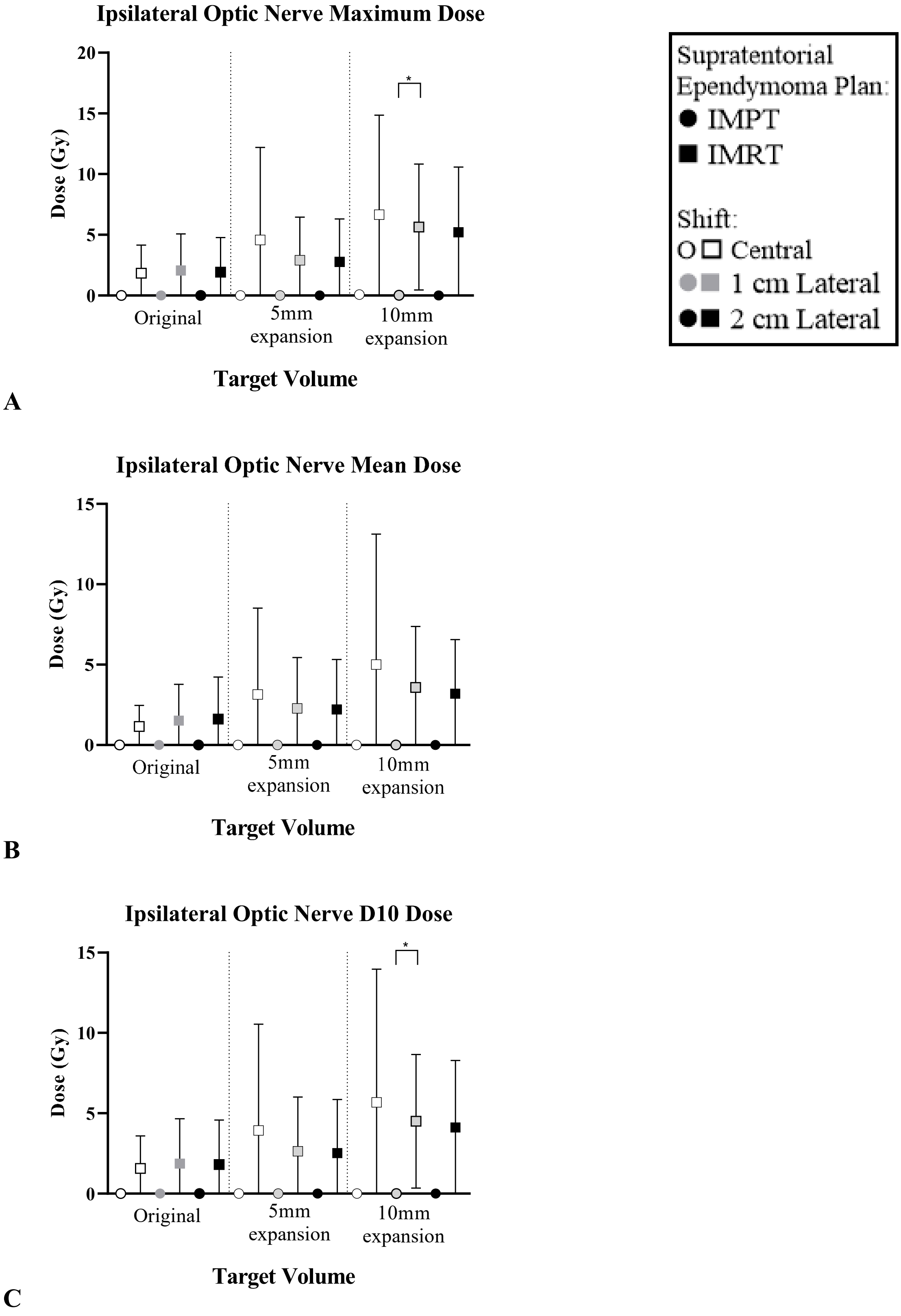

Figure S11. Average dose to ipsilateral optic nerve across six patients across IMPT and IMRT plans for three STEP volumes and locations. (**A**) Maximum dose. (**B**) Mean dose. (**C**) Dose to 10% of the structure. Paired T-test error bars represent the 95% confidence interval of the mean. * $p < 0.05$, ** $p < 0.01$, *** $p < 0.001$, **** $p < 0.0001$.

S12: PITUITARY GLAND RESULTS

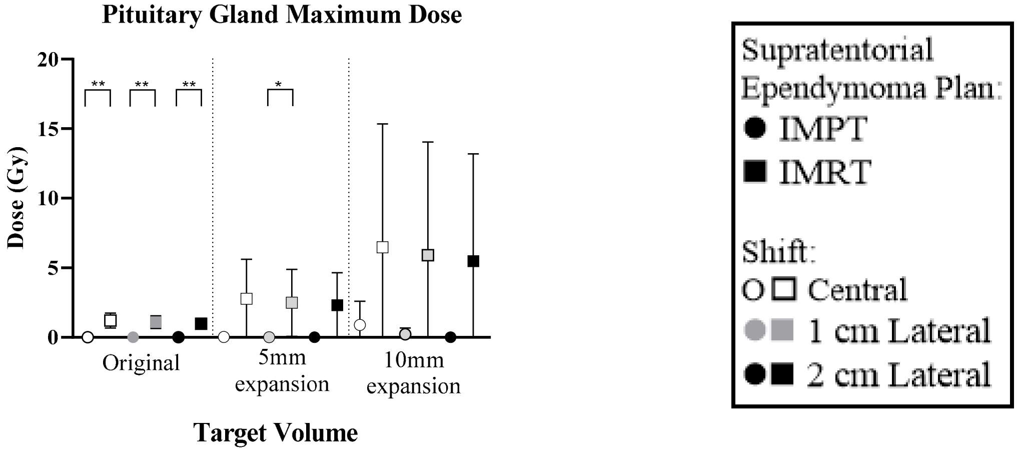

A

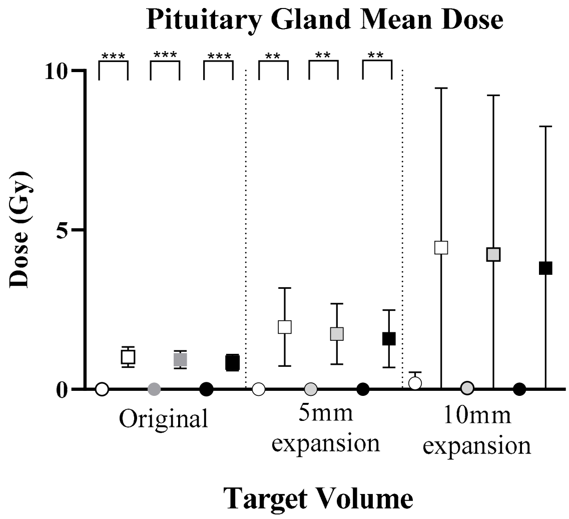

B

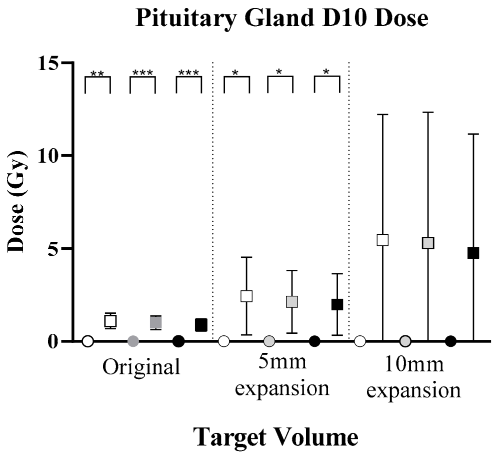

C

Figure S12. Average dose to pituitary gland across six patients across IMPT and IMRT plans for three STEP volumes and locations. (**A**) Maximum dose. (**B**) Mean dose. (**C**) Dose to 10% of the structure. Paired T-test error bars represent the 95% confidence interval of the mean. * $p < 0.05$, ** $p < 0.01$, *** $p < 0.001$, **** $p < 0.0001$.

Supplementary Figure S13. 13-year-old male head position for CT simulation compared to the rest of the cohort.

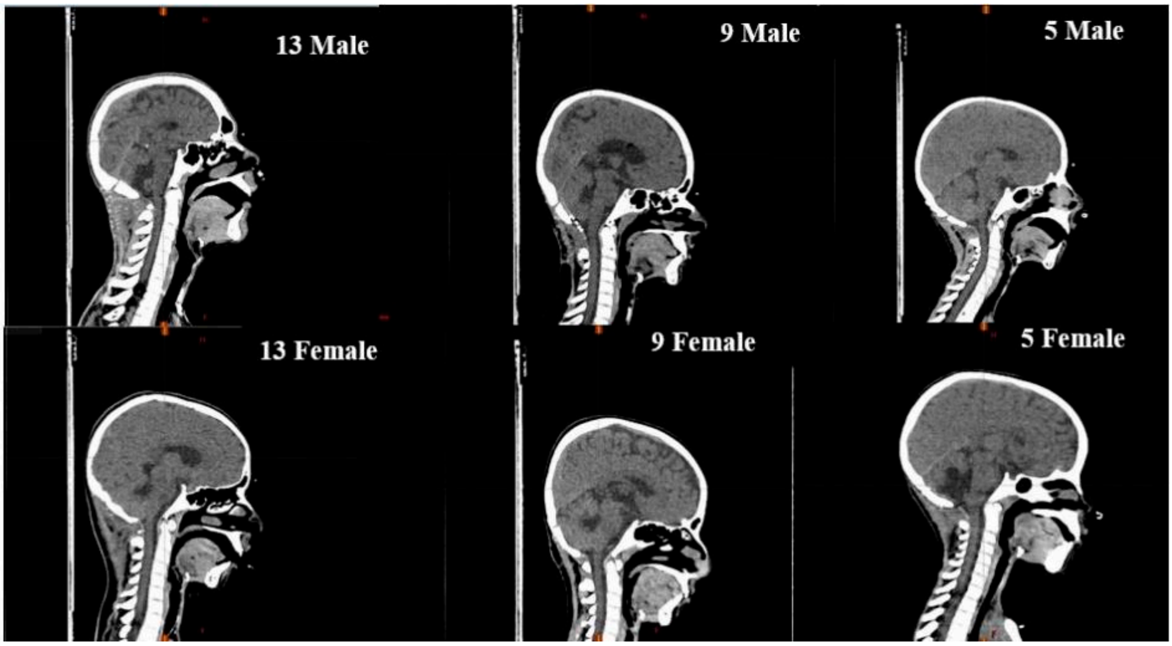

Figure S13. 13-year-old male head position for CT simulation compared to the rest of the cohort.

Supplementary Figure S14. Ipsilateral eye, lens and optic nerve sparing across IMPT (left) and IMRT (right) plans for STEP clinical volumes in 13-year-old female patient.

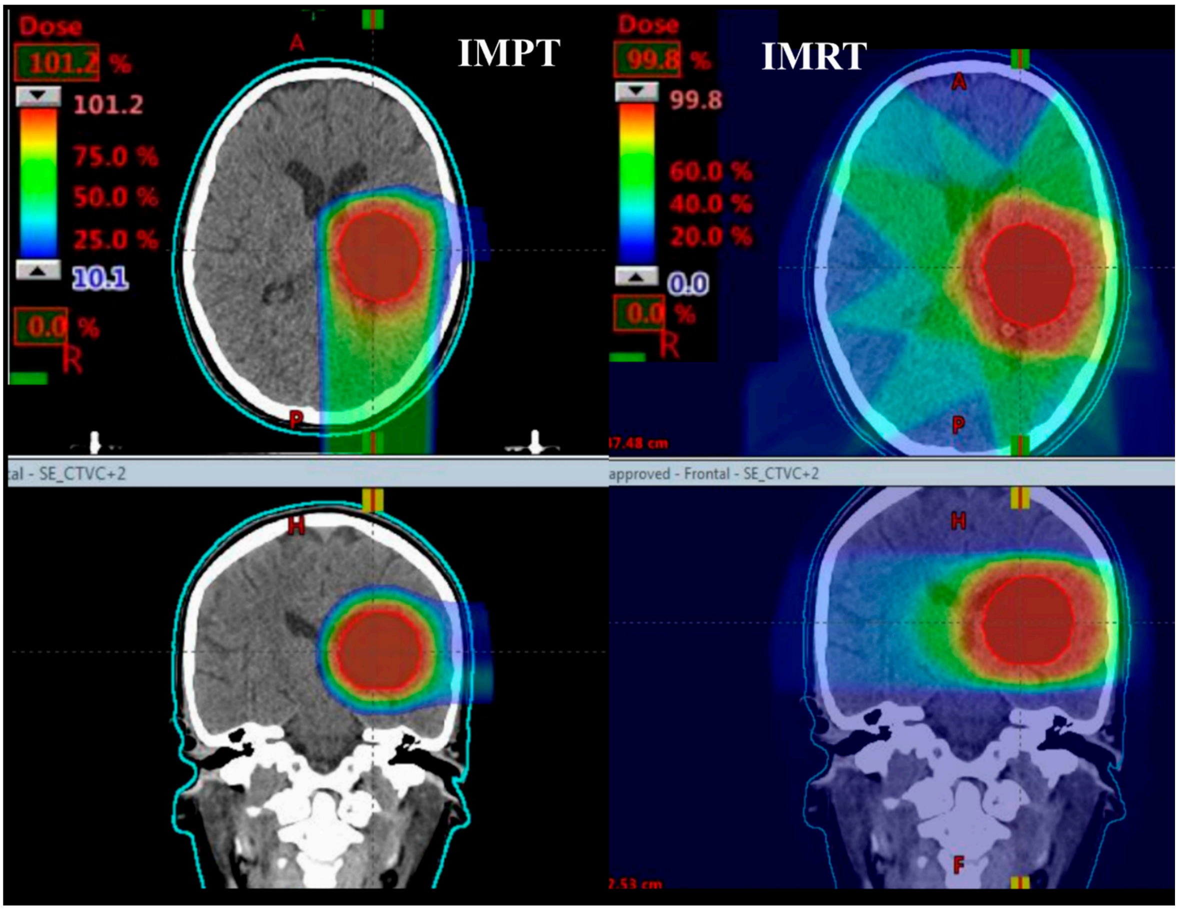

Figure S14. Ipsilateral eye, lens and optic nerve sparing across IMPT (left) and IMRT (right) plans for STEP clinical volumes in 13-year-old female patient.

Table S1. The absolute dose ranges for IMPT (Gy(RBE)) plans (grey shading) and IMRT (Gy) plans (white) for critical structures averaged across 6 patient plans collected for supratentorial ependymoma (prescribed dose 54 Gy/Gy(RBE)).

Structure	Dose Parameter (Gy)	1 cm Diameter			2 cm Diameter			3 cm Diameter		
		**Central**	**1 cm LAT**	**2 cm LAT**	**Central**	**1 cm LAT**	**2 cm LAT**	**Central**	**1 cm LAT**	**2 cm LAT**
Brainstem	Mean	1.1 (0.4–3)	0.4 (0.1–1.2)	0 (0–0.1)	3.5 (2.1–6.4)	1.8 (1–4.1)	0.4 (0.1–0.9)	7.7 (4.9–11.7)	4.1 (2.5–6.6)	1.3 (0.7–2.2)
		2.6 (1.4–5.1)	2 (1.2–3.9)	1.5 (1–2.8)	7.1 (4.4–11.2)	5.6 (3.5–9)	4.2 (2.6–6.7)	13.5 (10.4–17.5)	11.5 (9.3–15.5)	8.9 (7.2–12)
	D10	3.5 (0.8–11.7)	0.5 (0.1–2.2)	0.1 (0.1–0.1)	14.1 (7.3–27.8)	4.8 (1.7–15.2)	0.3 (0.1–0.7)	32.4 (19.1–47.7)	15.6 (7.9–27.2)	2.1 (0.7–5.1)
		7.1 (2.9–19.5)	5.2 (2.5–13.1)	3.9 (2–9.4)	27.5 (12.6–46.9)	19.7 (9–35.6)	13.5 (6.3–23.2)	48.6 (43.5–53)	41.1 (34.8–50.9)	29.2 (23.1–39.9)
Optic Chiasm	Mean	0.1 (0–0.4)	0 (0–0.2)	0 (0–0)	1.4 (0–3.5)	0.6 (0–1.8)	0.1 (0–0.4)	6.2 (0–13.1)	2.9 (0–7.4)	0.6 (0–1.9)
		2.9 (1.1–9.1)	2.5 (1–7.7)	2.3 (0.9–7.5)	8.1 (2–24.6)	6.8 (1.8–20.7)	5.9 (1.6–18.3)	18 (5.7–35.7)	15.3 (4.5–30)	13.2 (3.9–26.4)
	D10	0.3 (0–1)	0.1 (0–0.2)	0 (0–0.1)	4.7 (0–12)	2 (0–5.1)	0.1 (0–0.5)	17.6 (0.1–40.3)	11.7 (0–31.8)	1.7 (0–5.3)
		4 (1.3–11.7)	3.5 (1.2–10)	3.3 (1.1–9.7)	14.3 (2.6–34.2)	11.7 (2.2–28.8)	10.3 (2–26.3)	31.5 (9.4–50.4)	26.5 (7.7–46)	21.3 (6.2–33.2)
Ipsilateral Cochlea	Mean	0 (0–0)	0 (0–0)	0 (0–0)	0 (0–0)	0 (0–0)	0 (0–0)	0 (0–0)	0 (0–0)	0 (0–0)
		0.6 (0.5–0.6)	0.6 (0.5–0.7)	0.6 (0.5–0.7)	0.9 (0.8–1)	0.9 (0.8–1)	0.9 (0.8–1.1)	1.4 (1.2–1.5)	1.4 (1.3–1.6)	1.4 (1.2–1.6)
	D10	0 (0–0)	0 (0–0)	0 (0–0)	0 (0–0)	0 (0–0)	0 (0–0)	0 (0–0)	0 (0–0)	0 (0–0)
		0.6 (0.5–0.6)	0.6 (0.5–0.7)	0.6 (0.5–0.7)	1 (0.9–1)	1 (0.9–1.1)	1 (0.9–1.1)	1.5 (1.3–1.6)	1.5 (1.3–1.7)	1.5 (1.3–1.7)
Ipsilateral Eye	Mean	0 (0–0)	0 (0–0)	0 (0–0)	0 (0–0)	0 (0–0)	0 (0–0)	0 (0–0)	0 (0–0)	0 (0–0)
		1 (0.4–3.6)	1 (0.4–3.6)	1.4 (0.4–5.5)	2.2 (0.6–9.1)	1.8 (0.6–6.6)	2 (0.6–6.8)	3.9 (0.9–15.5)	3.1 (0.9–10.3)	2.8 (0.9–7.4)
	D10	0 (0–0)	0 (0–0)	0 (0–0)	0 (0–0)	0 (0–0)	0 (0–0)	0 (0–0)	0 (0–0)	0 (0–0)
		2.1 (0.4–10)	1.6 (0.4–6.5)	1.9 (0.4–7)	3.8 (0.7–17)	2.7 (0.7–8.2)	2.8 (0.8–7.4)	5.8 (1.1–21.9)	5.5 (1.2–19.7)	3.9 (1.2–8.1)
Ipsilateral Lens	Mean	0 (0–0)	0 (0–0)	0 (0–0)	0 (0–0)	0 (0–0)	0 (0–0)	0 (0–0)	0 (0–0)	0 (0–0)
		0.5 (0.3–1.3)	1 (0.3–3.9)	1.4 (0.3–6.1)	1.2 (0.5–4)	1.6 (0.5–6.2)	1.8 (0.5–6.5)	3.2 (0.8–14)	2.5 (0.8–7)	2.7 (0.8–6.7)
	D10	0 (0–0)	0 (0–0)	0 (0–0)	0 (0–0)	0 (0–0)	0 (0–0)	0 (0–0)	0 (0–0)	0 (0–0)
		0.6 (0.3–1.5)	1.3 (0.3–5.5)	1.4 (0.3–6.3)	1.5 (0.5–5.5)	1.7 (0.5–6.4)	2.1 (0.5–6.6)	4.2 (0.8- 18.9)	2.8 (0.8- 7.5)	2.8 (0.8–6.9)
Ipsilateral Optic Nerve	Mean	0 (0–0)	0 (0–0)	0 (0–0)	0 (0–0)	0 (0–0)	0 (0–0)	0 (0–0)	0 (0–0)	0 (0–0)
		1.2 (0.6–3.7)	1.5 (0.6–5.9)	1.6 (0.6–6.7)	3.1 (1–13.6)	2.3 (1–8.4)	2.2 (0.9–8.3)	5 (1.6–20.8)	3.6 (1.5–10.7)	3.2 (1.4–9.6)
	D10	0 (0–0)	0 (0–0)	0 (0–0)	0 (0–0)	0 (0–0)	0 (0–0)	0 (0–0)	0 (0–0)	0 (0–0)
		1.6 (0.7–5.5)	1.9 (0.7–7.3)	1.8 (0.7–7.2)	3.9 (1.3–16.8)	2.6 (1.3–9.2)	2.5 (1.2–9)	5.7 (2.2–21.8)	4.5 (2–12.2)	4.1 (1.9–12)
Pituitary Gland	Mean	0 (0–0)	0 (0–0)	0 (0–0)	0 (0–0)	0 (0–0)	0 (0–0)	0.2 (0–0.9)	0 (0–0.2)	0 (0–0)
		1 (0.8–1.6)	0.9 (0.7–1.5)	0.8 (0.7–1.3)	2 (1.3–4.3)	1.7 (1.2–3.6)	1.6 (1.1–3.3)	4.4 (2.1–14.2)	4.2 (1.9–13.9)	3.8 (1.7–12.5)
	D10	0 (0–0)	0 (0–0)	0 (0–0)	0 (0–0)	0 (0–0)	0 (0–0)	0 (0–0)	0 (0–0)	0 (0–0)
		1.1 (0.8–1.9)	1 (0.8–1.7)	0.9 (0.7–1.5)	2.4 (1.4–6.5)	2.1 (1.3–5.4)	2 (1.2–5.2)	5.5 (2.3–18.6)	5.3 (2.1–19)	4.8 (1.9–17.2)

Key: D10; dose received by 10% of the structure volume, mean; mean dose received by the structure, central; central target volume, SUP; superior shift of target volume, INF; inferior shift of target volume, LAT; lateral shift of target volume.

Table S2. The absolute dose ranges for IMPT (Gy(RBE)) plans (grey shading) and IMRT (Gy) plans (white) for critical structures averaged across 6 patient plans collected for infratentorial medulloblastoma (prescribed dose of 54 Gy/Gy(RBE) (23.4 Gy/Gy(RBE) craniospinal irradiation with 30.6 Gy/Gy(RBE) boost)).

Structure	Dose Parameter (Gy)	1 cm Diameter			0.5 cm Diameter			1.5 cm Diameter		
		**Central**	**1 cm SUP**	**1 cm INF**	**Central**	**1 cm SUP**	**1 cm INF**	**Central**	**1 cm SUP**	**1 cm INF**
Brainstem	Mean	38 (39.5–37.9)	35.6 (36.4–32.8)	32.7 (34.5–30.7)	30 (30.1–28.2)	30.1 (28.7–25.9)	28.4 (29.6–26.5)	37.3 (40.9–37.3)	38.9 (40.9–37.8)	33.1 (37.5–31.6)
		44.6 (46.1–42.8)	45.6 (45.7–44.2)	40.2 (43.1–36.1)	40 (42.3–37.9)	42.2 (41.3–38.3)	35.2 (39.1–32.1)	45.9 (47.4–44.3)	47.2 (47.7–46.8)	42.6 (45.4–39.3)
	D10	52.7 (52.4–52.9)	51.8 (48.2–52.9)	52 (51.1–53.1)	45 (40.7–50.1)	41.6 (33–47.6)	44.7 (37.7–51.6)	52.5 (51.9–53)	52.7 (51.8–53.1)	51.8 (49.3–53.1)
		53.7 (53.2–53.9)	53.3 (52.5–53.9)	53.5 (53.2–53.7)	52.9 (51.8–53.8)	51.7 (48.8–53.5)	52.7 (51.9–53.8)	53.1 (52.3–53.4)	53.3 (52.6–53.6)	53.3 (52.7–53.7)
Optic Chiasm	Mean	22.8 (22.8–22.9)	22.8 (22.8–22.9)	22.8 (22.8–22.9)	22.8 (22.8–22.9)	22.8 (22.8–22.9)	22.8 (22.8–22.9)	22.8 (22.8–22.9)	22.8 (22.8–22.9)	22.8 (22.8–22.9)
		30.7 (25.2–33.3)	32.3 (29.7–34.2)	28.9 (24.8–32.6)	28.9 (24.8–31.6)	29.9 (26.7–31.5)	27 (24.3–30.6)	33.4 (26.6–37.6)	35.4 (32.3–38)	31.4 (25–35.8)
	D10	23 (22.9–23)	23 (22.9–23)	23 (22.9–23)	23 (22.9–23)	23 (22.9–23)	23 (22.9–23)	23 (22.9–23)	23 (22.9–23)	23 (22.9–23)
		32.4 (25.7–35.9)	34.8 (31.9–36.8)	30.5 (25–35.6)	30.5 (25.1–35.5)	31.5 (27.8–35.2)	28.2 (24.5–33.6)	35.5 (27.8–40.4)	37.7 (35.9–40.6)	33.2 (25.3–37.1)
Ipsilateral Cochlea	Mean	22.7 (20.7–24.5)	22 (20.6–22.9)	22.4 (20.7–23)	21.7 (20.4–22.7)	21.7 (20.5–22.7)	21.7 (20.4–22.8)	23 (20.9–25.2)	22.5 (20.7–25.4)	22.5 (21.3–23.3)
		35.5 (34.9–35.9)	34.4 (32.8–36)	35 (34–35.7)	35.1 (33.9–35.7)	32 (25.7–35.6)	34.8 (33.9–35.7)	35.6 (35.1–36)	35.7 (35.2–36.1)	35.2 (34.6–35.9)
	D10	24.2 (22.7–28.2)	23.3 (22.6–24.9)	23.5 (22.7–24.9)	22.7 (21.9–23.1)	22.7 (21.9–23.1)	22.7 (21.9–23.2)	24.4 (23–27)	23.9 (22.7–26.9)	23.5 (22.9–25.3)
		36.3 (35.7–36.7)	35.3 (33.5–37.1)	35.8 (35–36.8)	35.6 (34.4–36.2)	32.9 (26.4–36)	33.7 (26.1–36.1)	36.6 (36.1–37.1)	36.7 (35.8–37.2)	36.2 (35.7–36.6)
Ipsilateral Eye	Mean	4.7 (2.3–7)	4.7 (2.3–7)	4.7 (2.3–7)	4.7 (2.3–7)	4.7 (2.3–7)	4.7 (2.3–7)	4.7 (2.3–7)	4.7 (2.3–7)	4.7 (2.3–7)
		10 (4.5–14)	9.7 (4.7–14.1)	10.1 (4.4–14.2)	8.7 (4.4–12.6)	8.3 (4.4–12.4)	8.6 (4.3–12.3)	12.4 (4.6–17.6)	12.2 (5.4–17.1)	11.6 (4.4–17.3)
	D10	10.7 (5.3–16)	10.7 (5.3–16)	10.7 (5.3–16)	10.7 (5.3–16)	10.7 (5.3–16)	10.7 (5.3–16)	10.7 (5.3–16)	10.7 (5.3–16)	10.7 (5.3–16)
		18.1 (8.3–24.3)	18.2 (8.3–24.9)	18 (8.1–24.7)	16.9 (8.1–22.7)	16.5 (8.2–23.1)	16.8 (8.1–22.6)	20.4 (8.3–28.1)	20.8 (8.6–28.2)	19.7 (8.2–27)
Ipsilateral Lens	Mean	1.9 (0.4–3)	1.9 (0.4–3)	1.9 (0.4–3)	1.9 (0.4–3)	1.9 (0.4–3)	1.9 (0.4–3)	1.9 (0.4–3)	1.9 (0.4–3)	1.9 (0.4–3)
		5.3 (2.7–7.7)	5 (2.8–7.5)	5.4 (2.6–7.5)	4.6 (2.6–7)	4.5 (2.6–7)	4.5 (2.5–6.9)	7.8 (2.8–10.9)	7 (3–10)	7.1 (2.7–11.2)
	D10	2.8 (0.6–4.9)	2.8 (0.6–4.9)	2.8 (0.6–4.9)	2.8 (0.6–4.9)	2.8 (0.6–4.9)	2.8 (0.6–4.9)	2.8 (0.6–4.9)	2.8 (0.6–4.9)	2.8 (0.6–4.9)
		7.2 (2.9–10.9)	6.7 (3–10.7)	7.8 (2.8–10.7)	6.2 (2.8–10.2)	6.1 (2.9–10.1)	6.1 (2.8–10)	10.1 (3–14.2)	9.1 (3.2–13.5)	9.5 (2.9–14.9)
Ipsilateral Optic Nerve	Mean	17.8 (15.4–20)	17.8 (15.4–20)	17.8 (15.4–20)	17.8 (15.4–20)	17.8 (15.4–20)	17.8 (15.4–20)	17.8 (15.4–20)	17.8 (15.4–20)	17.8 (15.4–20)
		24.8 (15.5–28.6)	25.5 (16.3–29.2)	23.9 (15.3–28.2)	22 (15.3–24.6)	21.8 (15.4–24.9)	21.3 (15.2–24)	28.3 (15.7–33.4)	29.3 (18.8–33.1)	26.3 (15.4–32.3)
	D10	22.3 (20.7–23.1)	22.3 (20.7–23.1)	22.3 (20.7–23.1)	22.3 (20.7–23.1)	22.3 (20.7–23.1)	22.3 (20.7–23.1)	22.3 (20.7–23.1)	22.3 (20.7–23.1)	22.3 (20.7–23.1)
		30.5 (23.9–33.9)	32 (25.9–34)	29.5 (23.7–33.9)	27.7 (23.7–30.6)	27.7 (23.8–29.6)	26.2 (23.4–30.1)	33.5 (24.2–37.2)	34.7 (28.7–36.5)	31.8 (23.7–37)
Pituitary Gland	Mean	22.8 (22.3–22.9)	22.8 (22.3–22.9)	22.8 (22.3–22.9)	22.8 (22.3–22.9)	22.8 (22.3–22.9)	22.8 (22.3–22.9)	22.8 (22.3–22.9)	22.8 (22.3–22.9)	22.8 (22.3–22.9)
		31.3 (28.1–33.6)	32.1 (30.8–34.1)	29.7 (24.8–33.9)	29.3 (25.8–31.2)	29.8 (29.6–30.1)	28 (24.4–30.2)	34.5 (30.3–37.2)	36 (34.1–38.3)	32.2 (26.1–35.8)
	D10	23 (22.9–23)	23 (22.9–23)	23 (22.9–23)	23 (22.9–23)	23 (22.9–23)	23 (22.9–23)	23 (22.9–23)	23 (22.9–23)	23 (22.9–23)
		32.3 (29–35.1)	33.6 (32–35.8)	30.7 (25–35.6)	30.1 (26.8–34.2)	30.2 (29.9–30.5)	28.5 (24.5–31.8)	35.7 (31.7–38.1)	37.2 (35.4–40.2)	33.1 (27–36.5)

Key: D10; dose received by 10% of the structure volume, mean; mean dose received by the structure, central; central target volume, SUP; superior shift of target volume, INF; inferior shift of target volume, LAT; lateral shift of target volume.

Table S3. Summary of T-test observations comparing significant differences between IMPT and IMRT plans for a range of structures and their respectively collected DVH metrics (maximum, mean, median and D10).

**Structure**	**Dosimetric Parameter (Gy)**	**Infratentorial Tumour Location (Medulloblastoma) Plan Comparison Observations**	**Supratentorial Tumour Location (Ependymoma) Plan Comparison Observations**
Brainstem	Maximum	No significant difference.	Reduction as target shifts laterally, however at a 3 cm diameter the IMPT dose does not change much.
	Mean	Significant difference - no real trends across volume/shift.	Lateral shift and expanding target volume show higher irradiation.
	Median	Significant difference in D10 and median dose for contracted target and superior shift compared to inferior shift.<br><br>Median dose was less significant the expanded the target. D10 was less significant for the expanded target.	Central target and expanding target volumes show higher irradiation.
	D10		Lateral shift and expanding target volume show higher irradiation.
Optic Chiasm	Maximum	Superior shift shows greater significant difference.	Central target and expanding target volume show higher irradiation.<br>Target size increases proportionally with significance and reduced irradiation in IMPT plan.
	Mean	Expansion target and superior shifts show greater significant difference.	
	Median		
	D10	Superior shifts show greater significant difference.	
Ipsilateral Cochlea	Maximum	Significant difference - no real trends across volume/shift.	Minimal IMPT dose. Irradiation increases with target volume.
	Mean		
	Median		Lateral shift and expanding target volumes show higher irradiation.
	D10		Irradiation increases with target volume.
Ipsilateral Eye	Maximum	Superior shifted targets receive higher irradiation.	Central target and expanding target volume show higher irradiation. Only significant for 3 cm diameter target (across all shifts).
	Mean	Contraction target receives reduced irradiation.	Central target and expanding target volume show higher irradiation.

		No pattern for shift.	
	Median	Contraction target receives reduced irradiation.	Expanding target volumes show higher irradiation.
	D10	Expanded targets receive higher irradiation.	
Ipsilateral Lens	Maximum	Large dose difference for expanded target.	Expanding target volume show higher irradiation.
	Mean		
	Median		
	D10	Expanded volume was significantly higher, contracted volume shows no impact on dose (across all locations).	
Ipsilateral Optic Nerve	Maximum	Large dose for expanded target.	Central target and expanding target volume show higher irradiation.
	Mean	Expanded volume and superior shifts are significantly higher, contracted volume shows no impact on dose (across all locations).	Expanding target volume show higher irradiation.
	Median		
	D10	Superior shift was significantly higher, contracted volume was significantly lower dose.	Central target and expanding target volume show higher irradiation.
Pituitary Gland	Maximum	Superior shift was significantly higher, contracted volume was significantly lower dose.	Central target and expanding target volume show higher irradiation.
	Mean		Central target and expanding target volume show higher irradiation. Target volume increases proportionally with significance and reduced irradiation in IMPT plan.
	Median		Central target and expanding target volume show higher irradiation.
	D10	Superior shift was significantly higher, expanded volume was significantly higher dose.	
**Key:** Mean; mean dose received by the structure, maximum; maximum dose received by the structure, median; dose received by 50% of the structure volume, D10; dose received by 10% of the structure volume.			

Table S4. Mean and D10 dose difference (Δ IMRT (Gy)–IMPT (Gy(RBE))) for critical structures across 3 patient ages and location (volumes averaged). Prescribed dose for both tumour sites was 54 Gy/Gy(RBE) (23.4 Gy/Gy(RBE) craniospinal irradiation with 30.6 Gy/Gy(RBE) boost for medulloblastoma).

Structure	Dosimetric Parameter (Gy)	Infratentorial Tumour Location (Medulloblastoma)									Supratentorial Tumour Location (Ependymoma)								
		5-Year-Old Male and Female			9-Year-Old Male and Female			13-Year-Old Male and Female			5-Year-Old Male and Female			9-Year-Old Male and Female			13-Year-Old Male and Female		
		Central	1 cm SUP	1 cm INF	Central	1 cm SUP	1 cm INF	Central	1 cm SUP	1 cm INF	Central	1 cm LAT	2 cm LAT	Central	1 cm LAT	2 cm LAT	Central	1 cm LAT	2 cm LAT
Brainstem	Δ Mean	7.8	8.8	7.8	8.4	9.0	8.2	8.9	10.8	9.9	4.0	5.0	5.1	3.4	3.9	3.9	3.4	3.8	3.8
	Δ D10	3	2.9	4.7	3.0	4.4	2.2	3.3	4.9	4.0	11.3	16.9	17.9	10.9	14.2	13.0	11.0	13.9	13.1
Optic Chiasm	Δ Mean	8.0	10.1	5.03	9.4	9.7	7.6	7.0	9.2	6.0	4.3	4.6	4.6	5.0	4.7	4.2	11.8	11.6	11.8
	Δ D10	9.4	11.8	6.4	11.3	11.5	9.2	8.7	11.8	7.3	6.5	7.0	8.2	8.5	8.0	7.7	12.0	12.7	16.9
Ipsilateral Cochlea	Δ Mean	12.6	13.1	12.9	13.7	11.4	13.1	12.3	11.1	12.3	0.9	0.9	0.9	0.9	0.9	0.9	0.9	1.0	1.0
	Δ D10	12	12.7	10.9	12.7	10.9	12.6	12.4	11.3	12.4	1	1.0	1	0.9	1	1	1.0	1.0	1.0
Ipsilateral Eye	Δ Mean	6.6	7.3	6.3	6.4	5.5	6.2	3.7	3.1	3.5	1.3	1.5	1.9	0.6	0.6	0.6	5.0	3.7	3.6
	Δ D10	7.9	8.8	7.6	9.7	9.3	9.4	5.6	5.1	5.3	2.3	2.8	3.5	0.7	0.8	0.8	8.5	6.1	4.1
Ipsilateral Lens	Δ Mean	4.9	5.0	4.7	4.3	3.6	4.4	2.8	2.2	2.2	0.9	1.3	1.7	0.5	0.5	0.5	3.4	3.1	3.4
	Δ D10	6.6	6.7	6.5	5.2	4.4	5.6	3.1	2.4	2.9	1.0	1.7	2.2	0.5	0.5	0.5	4.5	3.5	3.5
Ipsilateral Optic Nerve	Δ Mean	7.8	9.7	6.3	9.2	8.8	7.8	4.6	4.6	4.0	1.2	1.5	1.3	1.0	1.0	1.0	6.8	4.7	4.6
	Δ D10	8.4	10.6	6.3	9.8	9.8	8.2	6.6	7.0	5.9	1.5	1.9	1.7	1.4	1.4	1.2	8.1	5.6	5.4
Pituitary Gland	Δ Mean	9.0	10.5	6.7	9.3	9.4	8.3	8.4	9.4	6.5	1.6	1.4	1.3	1.5	1.4	1.3	4.0	3.9	3.5
	Δ D10	9.6	11.5	7.2	10.0	10.3	8.9	9.5	10.2	7.1	1.8	1.6	1.4	1.7	1.5	1.4	5.4	5.2	4.7

Key: Δ; dose difference (IMRT-IMPT), D10; dose received by 10% of the structure volume, mean; mean dose received by the structure, central; central target volume, SUP; superior shift of target volume, INF; inferior shift of target volume, LAT; lateral shift of target volume.
